# Supplementary material for: Prevalence of and factors associated with multimorbidity among 18 101 adults in the South East Asia Community Observatory Health and Demographic Surveillance System in Malaysia: a population-based, cross-sectional study of the MUTUAL consortium
Source: BMJ Open. 2022 Dec 23;12(12):e068172. doi: 10.1136/bmjopen-2022-068172 (PMC9791377; doi:10.1136/bmjopen-2022-068172)
Supplement: Author's manuscript [file bmjopen-2022-068172.draft_revisions.pdf]

BMJ Open

BMJ Open is committed to open peer review. As part of this commitment we make the peer review history of every article we publish publicly available.

When an article is published we post the peer reviewers' comments and the authors' responses online. We also post the versions of the paper that were used during peer review. These are the versions that the peer review comments apply to.

The versions of the paper that follow are the versions that were submitted during the peer review process. They are not the versions of record or the final published versions. They should not be cited or distributed as the published version of this manuscript.

BMJ Open is an open access journal and the full, final, typeset and author-corrected version of record of the manuscript is available on our site with no access controls, subscription charges or pay-per-view fees (http://bmjopen.bmj.com).

If you have any questions on BMJ Open's open peer review process please email info.bmjopen@bmj.com

BMJ Open

Prevalence and factors associated with multimorbidity in 18,101 adults in Malaysia: A population-based study of the MUTUAL consortium

Journal:	*BMJ Open*
Manuscript ID	bmjopen-2022-068172
Article Type:	Original research
Date Submitted by the Author:	10-Sep-2022
Complete List of Authors:	Tan, Michelle; King's College London Institute of Psychiatry Psychology and Neuroscience, Department of Health Service and Population Research; Monash University Faculty of Medicine Nursing and Health Sciences, 4 Victorian Heart Institute<br>Prina, A.Matthew; King's College London Institute of Psychiatry Psychology and Neuroscience, Department of Health Service and Population Research,<br>Muniz Terrera, Graciela; University of Edinburgh UK; Ohio University, Department of Primary Care<br>Mohan, Devi; Monash University Malaysia, Global Public Health. Jeffrey Cheah School of Medicine and Health Sciences<br>Ismail, Roshidi; Monash University Malaysia, SEACO<br>Assefa, Esubalew; Centre for Innovative Drug Development and Therapeutic Trials for Africa; Jimma University, Department of Economics<br>Keinert, Ana; Universidade Federal de São Paulo, Departamento de Psiquiatria e Psicologia Médica<br>bin Kassim, Zaid; Segamat District Health Office<br>Allotey, P; United Nations University International Institute for Global Health, IIGH; World Health Organization (WHO), Department of Sexual and Reproductive Health and Research<br>Reidpath, Daniel; Monash University Malaysia, Jeffrey Cheah School of Medicine and Health Sciences; International Centre for Diarrhoeal Disease Research Bangladesh<br>Su, Tin ; Monash University - Malaysia Campus
Keywords:	EPIDEMIOLOGY, HEALTH SERVICES ADMINISTRATION & MANAGEMENT, PUBLIC HEALTH

SCHOLARONE™
Manuscripts

I, the Submitting Author has the right to grant and does grant on behalf of all authors of the Work (as defined in the below author licence), an exclusive licence and/or a non-exclusive licence for contributions from authors who are: i) UK Crown employees; ii) where BMJ has agreed a CC-BY licence shall apply, and/or iii) in accordance with the terms applicable for US Federal Government officers or employees acting as part of their official duties; on a worldwide, perpetual, irrevocable, royalty-free basis to BMJ Publishing Group Ltd ("BMJ") its licensees and where the relevant Journal is co-owned by BMJ to the co-owners of the Journal, to publish the Work in this journal and any other BMJ products and to exploit all rights, as set out in our licence.

The Submitting Author accepts and understands that any supply made under these terms is made by BMJ to the Submitting Author unless you are acting as an employee on behalf of your employer or a postgraduate student of an affiliated institution which is paying any applicable article publishing charge ("APC") for Open Access articles. Where the Submitting Author wishes to make the Work available on an Open Access basis (and intends to pay the relevant APC), the terms of reuse of such Open Access shall be governed by a Creative Commons licence – details of these licences and which Creative Commons licence will apply to this Work are set out in our licence referred to above.

Other than as permitted in any relevant BMJ Author's Self Archiving Policies, I confirm this Work has not been accepted for publication elsewhere, is not being considered for publication elsewhere and does not duplicate material already published. I confirm all authors consent to publication of this Work and authorise the granting of this licence.

For peer review only

Full title:

Prevalence and factors associated with multimorbidity in 18,101 adults in Malaysia: A population-based study of the MUTUAL consortium

Short title:

Prevalence and risk factors for multimorbidity in a middle-income country: A population-based study

Michelle M. C. Tan[1,2,3,4], Matthew Prina[1], Graciela Muniz Terrera[5,6], Devi Mohan[2], Mohammad Roshidi Ismail[3], Esubalew Assefa[7,8,9], Ana Á. M. Keinert[10], Zaid Kassim[11], Pascale Allotey[2,12,13], Daniel Reidpath[3,14], and Tin Tin Su[2,3,4]

Author affiliations:

[1] Department of Health Service and Population Research, Institute of Psychiatry, Psychology & Neuroscience, King's College London, London, United Kingdom.
[2] Global Public Health, Jeffrey Cheah School of Medicine and Health Sciences, Monash University Malaysia, Sunway City, Selangor, Malaysia.
[3] South East Asia Community Observatory (SEACO), Jeffrey Cheah School of Medicine and Health Sciences, Monash University Malaysia, Sunway City, Selangor, Malaysia.
[4] Victorian Heart Institute, Clayton Campus, Faculty of Medicine, Nursing and Health Sciences, Monash University, Victoria, Australia.
[5] Edinburgh Dementia Prevention, University of Edinburgh and Western General Hospital, Edinburgh, Scotland, United Kingdom.
[6] Department of Social Medicine, Heritage College of Osteopathic Medicine, Ohio University, Ohio, United States.
[7] Centre for Innovative Drug Development and Therapeutic Trials for Africa (CDT-Africa), College of Health Sciences, Addis Ababa University, Addis Ababa, Ethiopia.
[8] Department of Economics, College of Business and Economics, Jimma University, Jimma, Ethiopia.
[9] Department of Economics, Faculty of Arts and Social Sciences, The Open University, Milton Keynes, United Kingdom.
[10] Departamento de Psiquiatria e Psicologia Médica, Escola Paulista de Medicina, Universidade Federal de São Paulo (UNIFESP), Rua Major Maragliano, Vila Mariana, São Paulo - SP, Brazil.
[11] District Health Office Segamat, Ministry of Health Malaysia, Johor, Malaysia.
[12] International Institute for Global Health, United Nations University, Kuala Lumpur, Malaysia.
[13] Department of Sexual and Reproductive Health and Research, World Health Organization (WHO), Geneva, Switzerland.
[14] International Centre for Diarrhoeal Disease Research, Bangladesh (icddr,b), Dhaka, Bangladesh.

For peer review only - http://bmjopen.bmj.com/site/about/guidelines.xhtml

***Correspondence to:**

Dr Michelle M.C. Tan, PhD
tan_mun.chieng@kcl.ac.uk / michelle.tanmunchieng@gmail.com

Dr Matthew Prina, PhD
matthew.prina@kcl.ac.uk

Prof Tin Tin Su, PhD
TinTin.Su@monash.edu

For peer review only

STRENGTHS AND LIMITATIONS OF THIS STUDY

- Most studies of the epidemiology of multimorbidity globally have focused on older populations in high-income countries (HICs), and data from younger adults and low- and middle-income countries (LMICs) are notably lacking. Evidence and knowledge concerning the trends and risk factors for multimorbidity remain limited in LMICs, including the multi-ethnic and rapidly ageing middle-income country, Malaysia.
- This is the first study that examined the epidemiology of multimorbidity of community-dwelling adults aged 18 and above on a large health and demographic surveillance system (HDSS) scale in Malaysia.
- Our findings facilitated the identification of high-risk populations, and multimorbidity was found to be not just an issue of the elderly.
- The magnitude and risk factors of multimorbidity determined in the diverse study population suggest the need to develop context-specific guidelines on diagnosing and managing multiple chronic conditions to ensure better health service provision, health management and resource deployment.

For peer review only - http://bmjopen.bmj.com/site/about/guidelines.xhtml

ABSTRACT

Objective: Evidence and knowledge concerning the trends and risk factors for multimorbidity remain limited. This study aimed to assess the prevalence of multimorbidity in a community-dwelling general adult population and explore its association with socio-economic and demographic factors on a large health and demographic surveillance system (HDSS) scale.

Methods: Of the 45,246 participants of the South East Asia Community Observatory (SEACO) database, 18,101 adults aged 18–97 years (mean age 47 years, 55.6% female) were included. A total of 13 chronic diseases were selected and were further classified into 11 medical conditions to account for multimorbidity. The conditions were heart disease, stroke, diabetes mellitus, hypertension, chronic kidney disease, musculoskeletal disorder, obesity, asthma, vision problem, hearing problem, and physical mobility problem. Multimorbidity was defined as the coexistence of two or more chronic conditions per individual. A multivariable logistic regression model was performed to identify factors associated with multimorbidity.

Results: Of the study cohort, 28.5% people lived with multimorbidity. The individual prevalence of the chronic conditions ranged from 1.0% to 24.7%, with musculoskeletal disorder (24.7%), obesity (20.7%) and hypertension (18.4%) as the most prevalent chronic conditions. The number of chronic conditions increased linearly with age ($p < 0.001$). In the logistic regression model, we observed that multimorbidity is associated with female sex (adjusted-OR=1.28), education levels (primary education compared to no education: adjusted-OR=0.64, secondary education: adjusted-OR=0.60, tertiary education: adjusted-OR=0.65), and employment status (working adults compared to retirees: adjusted-OR=0.70), in addition to age (adjusted-OR=1.05).

Conclusions: The current single-disease services in primary and secondary care should be accompanied by strategies to address complexities associated with multimorbidity, taking into account the factors associated with multimorbidity identified. Future research is needed to identify the most commonly occurring clusters of chronic diseases and their risk factors to develop more efficient and effective multimorbidity prevention and treatment strategies.

Keywords: Multimorbidity; epidemiology; prevalence; risk factors; low- and middle-income countries.

For peer review only - http://bmjopen.bmj.com/site/about/guidelines.xhtml

For peer review only

INTRODUCTION

Multimorbidity, as defined by the World Health Organization (WHO) (1), refers to the coexistence of two or more chronic health conditions in a single individual. The rising tide of multimorbidity is a key challenge for both patients and healthcare systems worldwide (2). Multimorbidity is associated with lower quality of life, impaired functional capacity, psychological distress, increased premature mortality, and greater healthcare service utilisation and medical expenditure (3-6).

There are numerous ways to measure multimorbidity, such as disease counts, conditions weighted or combined with other parameters, diagnostic categories, drug use, and physiological measures (7). Despite these various methods, a simple count of chronic diseases is the most popular method of measuring multimorbidity due to its ease of applying. In a recent systematic review and meta-analysis (8), it was found that the prevalence of multimorbidity is comparable between high-income countries (HICs) and low- and middle-income countries (LMICs). While multimorbidity affects all nations and age groups, most studies of the prevalence of multimorbidity have focused on older populations in HICs, and data from younger adults and LMICs are notably lacking (2). Similarly, factors that may increase the risk of multimorbidity are poorly understood, with ageing not being the only factor associated with high levels of multimorbidity (9). Consequently, it has not been possible to evaluate and develop patient-centred prevention and treatment strategies targeting multimorbidity and such risk factors. The COVID-19 pandemic further drew attention to the crucial contribution of multimorbidity to the need for sound public health measures and rapid identification of effective medical interventions grounded on risk stratification (10). To better manage the consequences of such an adverse phenomenon, a better understanding of the

For peer review only - http://bmjopen.bmj.com/site/about/guidelines.xhtml

prevalence and factors associated with multimorbidity among diverse study populations became essential.

According to the World Bank Classification (11), Malaysia is a high middle-income country in Asia, with a diverse population. After neighbouring Singapore, Malaysia is the second most urbanized country in South East Asia (12). While it is also experiencing a rapid demographic transition, edging towards population ageing and increasing prevalence of non-communicable diseases (12), there is limited information on multimorbidity of the general population in the real-world community setting. Very few studies have been able to evaluate the epidemiology of multimorbidity in Malaysia – the World Health Survey (13, 14) derived from multimorbidity measures that included a restricted number of conditions and relied on relatively old data. The only contextually-relevant studies by in-country researchers were either smaller in sample size (15) or included a limited number of chronic conditions (four and seven conditions, respectively) in the older population (16, 17).

This study is a response of the MUltimorbidity ThroUgh cApacity buiLding (MUTUAL) consortium, developed to better understand and address the growing challenge of multimorbidity in LMICs. The aims of this study were, therefore, to fill the evidence gaps by identifying the country-specific prevalence and factors associated with multimorbidity. To the best of our knowledge, this was the first study that examined in detail a community-dwelling general adult population on a large health and demographic surveillance system (HDSS) scale in Malaysia. To underline the complexity of multimorbidity, and to understand the epidemiology in different age groups, we considered a comprehensive range of chronic health conditions and did not restrict our study to the elderly.

METHODS

For peer review only - http://bmjopen.bmj.com/site/about/guidelines.xhtml

Data source and study population

Data for the analysis were drawn from the dynamic South East Asia Community Observatory (SEACO) HDSS database that was established in 2011 in Malaysia. The SEACO HDSS profile has been published previously (18), and is updated here. SEACO HDSS site has been capturing detailed longitudinal information related to health and diseases among individuals and households to improve the holistic experience in the local community and the wider population. SEACO remains the sole HDSS with International Standards Organization (ISO) certification for quality data collection and data management processes (ISO 9001:2015) among over 48 HDSS from LMICs worldwide (http://www.indepth-network.org/). Besides the MUTUAL consortium, SEACO is also a member of the International Network for the Demographic Evaluation of Populations and Their Health (INDEPTH) network and the recently-established HDSS-Asia network.

Comprehensive accounts of socio-demographic, lifestyle and medical information were gathered from all participants recruited to the population-wide baseline Core Census 2012 in an electronic handheld device-assisted personal interview administered by trained interviewers. There were 45,246 community-dwelling residents aged 0 100 from 13,431 households enrolled in the Core Census 2012 for baseline enumeration. Of the participants, 38,228 participants were ≥ 18 years old (n=31,595) with a mean age of 45 years (SD 16.9 years). In 2013-2014, the first five-yearly population-wide health survey, Health Round Wave 1, was undertaken at the individual level among 25,168 participants aged 5 years and above who were part of the core sample. An overview of the population's overall health was assessed, including medical history, mental health, well-being, quality of life, lifestyle factors, and health service utilisation. Of them, 18,101 adults were aged 18 and older [mean age 47.3 years (SD 16.3 years)]. Data from this cohort of Health Round Wave 1 (2013/2014) and the

For peer review only - http://bmjopen.bmj.com/site/about/guidelines.xhtml

Core Census 2012 were included in the current study for a longer list of chronic conditions to be considered. A flowchart of the population in this study is presented in Figure 1. Ethics approvals were obtained from the Monash University Human Research Ethics Committee (MUHREC CF11/3663 – 2011001930 and 2013-3837-3646). All participants had provided written consent prior to data collection.

Chronic condition measures and definition of multimorbidity

While chronic health conditions were self-reported and not directly based on formal clinical diagnoses, participants were asked whether they had ever been told by medical professionals that they had a particular chronic health condition based on a series of dichotomous questions (yes/no). The conditions were heart disease (including coronary artery disease, myocardial infarction, angina pectoris, congestive heart failure), stroke, type 1 and/or type 2 diabetes mellitus (DM), hypertension, chronic kidney disease, end-stage renal failure (ESRF) requiring dialysis, arthritis, joint pain, back pain, asthma, vision problem, hearing problem, and physical mobility problem. Additionally, obesity (BMI ≥ 30 kg/m^2) was included and defined using the principal body mass index (BMI) classification by the WHO (19), by which weight (in kilograms) was divided by height squared (in metres). Weight and height were estimated using electronic portable weighing scales with electronic height sensors (Patient Focus, model GBS-721), in which participant was barefoot and wearing light clothing during the measurement. Due to collinearity, participants who had any musculoskeletal disorder, either arthritis or joint pain or back pain or all three, were combined into one condition – 'arthritis, joint pain and/or back pain'. Likewise, the mutually exclusive chronic kidney disease and ESRF requiring dialysis were considered together as 'chronic kidney disease'. To avoid potential bias caused by spurious associations, medical conditions with a prevalence of ≤ 1%

For peer review only - http://bmjopen.bmj.com/site/about/guidelines.xhtml

were excluded, such as speech problem and gastrointestinal conditions. Acute infectious diseases consisting of dengue fever and urinary tract infection were also excluded.

Each of the included 11 chronic conditions was coded as binary, where presence=1 and absence=0. In the present study, multimorbidity is defined as the presence of two or more of these concurrent long-term physical conditions for each participant.

Mental health measures

Mental health was measured using the Depression, Anxiety and Stress Scale–21 (DASS-21) (20), the short form of the DASS-42 (21). It is a set of three self-report scales designed to measure the emotional states of depression, anxiety, and stress. Each of the three DASS-21 scales contains 7 items that are divided into subscales with similar content. The subscales correspond to the following diagnostic categories: 'Depression' for mood disorders, 'Anxiety' for panic disorder and 'Stress' for generalized anxiety disorder. The individual scale items were scored on a 4-point Likert scale (never, sometimes, often, and almost always) of frequency or severity of participants' experiences over the past week. The score of each subscale was summed and multiplied by two to be comparable to the long form of DASS. Higher scores indicate greater levels of symptoms. Participants with a cut-off score of ≥ 10 were classified as having depressive symptoms (21).

Study variables

Socio-demographic variables such as age (18–24, 25–34, 35–44, 45–54, 55–64, ≥ 65), sex (female or male), ethnicity (Malay, Chinese, Indian, other), education attainment (no formal education, primary, secondary, tertiary education), employment status (working/self-employed, homemaker, unemployed, retired/pension, student), personal monthly gross

For peer review only - http://bmjopen.bmj.com/site/about/guidelines.xhtml

income (< MYR 1,000, MYR 1,000-1,999, ≥ MYR 2,000), monthly household income [grouped into bottom 40% (B40), middle 40% (M40), top 20% (T20)], and marital status (categorised as married, never married, widowed/divorced/separated) were included. Private health insurance was coded into two categories (yes through self-subscription/employer, or no), and payer for health cost referred to either self/family member, government/pension, employer-provided health cover or personal health insurance. Health service utilisation included medication prescriptions in the last two weeks (i.e., antidiabetic and antihypertensive drugs, coded into yes or no), hospital admissions (≥ 1 night in the past 6 months, categorised as yes or no) and emergency department visits in the past 6 months (yes or no).

Data analysis

Descriptive statistics were used to summarise information regarding socio-demographic variables, clinical profiles, health service utilisation, and the prevalence of multimorbidity and individual chronic diseases. The age groups were compared using one-way ANOVA with post-hoc Bonferroni test or chi-square (X^2) test. The multimorbidity groups (yes or no) were compared using independent *t*-test or X^2 test. The total number of chronic conditions was counted and categorised as no multimorbidity (0-1) and multimorbidity (2, 3 and ≥ 4 chronic conditions, respectively). A multivariable logistic regression model using the enter method with entry criteria of α=0.05 and removal criteria=0.10 was performed to identify factors associated with multimorbidity (yes/no), controlled for socio-demographic covariates (age, sex, ethnicity, education level, and employment status). Covariates were selected, further categorised and included based on previous literature and theory. Diagnostic tests for complete case models were conducted. All analyses were performed using Stata/MP (Version

For peer review only - http://bmjopen.bmj.com/site/about/guidelines.xhtml

17, Stata Corp LP, College Station, TX, USA) and IBM SPSS Statistics (Version 28, Armonk, New York, USA). All statistical analyses were two-sided with a significance of $p < 0.05$.

Patient and public involvement statement

Neither the study participants nor the public were involved in designing, planning, conducting, reporting or disseminating our research.

RESULTS

Socio-economic and demographic characteristics of the study cohort are reported in Table 1. The average (range) age of the participants was 47 (18–97) years, 55.6% were females, two-thirds were Malays, and the majority were married. The study population largely drawn from a semi-rural state in Malaysia was mainly represented by subjects who had lower education, household and personal income levels (Table 1).

Table 1 Summary of population characteristics, showing socio-demographic characteristics (overall and by multimorbidity status) (n=18,101)

	**Overall (n=18,101)**	**Multimorbidity**		***p*-value**
		**Yes (n=4669) (28.5%)**	**No (n=11,688) (71.5%)**	
Age (years)				**< 0.001**
Mean±SD	47.3±16.3	55.3±13.8	44.0±15.8	
Sex, n (%)				**< 0.001**
Female	9089 (55.6%)	2824 (60.5%)	6265 (53.6%)	
Male	7268 (44.4%)	1845 (39.5%)	5423 (46.4%)	
Ethnicity, n (%)				**0.005**
Malay	10,741 (65.7%)	3025 (64.9%)	7716 (66.1%)	
Chinese	3586 (21.9%)	1100 (23.6%)	2486 (21.3%)	
Indian	1594 (9.8%)	419 (9.0%)	1175 (10.1%)	
Other	418 (2.6%)	119 (2.6%)	299 (2.6%)	
Marital status, n (%)				**< 0.001**

For peer review only - http://bmjopen.bmj.com/site/about/guidelines.xhtml

Never married	2584 (16.0%)	304 (6.5%)	2280 (19.8%)	
Married	11,846 (73.2%)	3568 (76.7%)	8278 (71.8%)	
Widowed/Divorced/Separated	1745 (10.8%)	781 (16.8%)	964 (8.4%)	
Education level, n (%)				**< 0.001**
No formal education	841 (5.2%)	314 (6.9%)	527 (4.6%)	
Primary	4842 (30.1%)	1324 (28.9%)	3518 (30.6%)	
Secondary	9012 (56.1%)	2529 (55.2%)	6483 (56.4%)	
Tertiary	1373 (8.5%)	411 (9.0%)	962 (8.4%)	
Employment status, n (%)				**< 0.001**
Working/Self-employed	8189 (50.3%)	1749 (37.6%)	6440 (55.4%)	
Homemaker	5017 (30.8%)	1704 (36.7%)	3313 (28.5%)	
Unemployed	1868 (11.5%)	743 (16.0%)	1125 (9.7%)	
Retired/Pension	885 (5.4%)	421 (9.1%)	464 (4.0%)	
Student	313 (1.9%)	32 (0.7%)	281 (2.4%)	
Personal gross monthly income (MYR)				**< 0.001**
Mean±SD	1204.0±1515.4	1094.7±1684.1	1224.0±1383.4	
< 1,000	8554 (52.3%)	2707 (58.0%)	5847 (50.0%)	
1,000-1,999	4575 (28.0%)	1107 (23.7%)	3468 (29.7%)	
≥ 2,000	3228 (19.7%)	855 (18.3%)	2373 (20.3%)	
Household monthly income (MYR)†				**< 0.001**
Mean±SD	3193.8±3537.1	2720.9±3333.0	3224.2±3357.7	
Bottom 40% (B40)	12,263 (75.0%)	3679 (78.8%)	8584 (73.4%)	
Middle 40% (M40)	3076 (18.8%)	749 (16.0%)	2327 (19.9%)	
Top 20% (T20)	1018 (6.2%)	241 (5.2%)	777 (6.6%)	
Private health insurance (Through employer/self-subscription), n (%)				**< 0.001**
Yes	3650 (22.6%)	955 (20.7%)	2695 (23.3%)	
No	12,507 (77.4%)	3652 (79.3%)	8855 (76.7%)	
Payer for healthcare cost, n (%)				**< 0.001**
Self/Family member	13,329 (82.0%)	3715 (80.1%)	9614 (82.8%)	
Government/Pension	2293 (14.1%)	805 (17.4%)	1488 (12.8%)	
Employer-provided health cover	492 (3.0%)	85 (1.8%)	407 (3.5%)	
Personal private health insurance	139 (0.9%)	32 (0.7%)	107 (0.9%)	

Abbreviations: SD=Standard deviation.
Numbers may not add up to totals due to missing data.
MYR=Malaysian Ringgit, MYR 1 was equivalent to the British Pound (GBP) £ 0.18 at the time of publication.
†Categorised as three income thresholds according to the local Department of Statistics, where B40, M40 and T20 represent groups with monthly income of < MYR 3860, MYR 3860–8319, and > MYR 8319, respectively.
Statistical significance testing by chi-square (χ^2) test and independent t-test.

Multimorbidity was identified in 28.5% of participants, with 15.9% having two conditions, and 12.6% having three or more conditions. See Table 2 for the clinical characteristics of the study cohort, including the prevalence of multimorbidity and individual chronic conditions. Approximately 44.3% of the cohort had no chronic condition, and 27.2% had one condition. The individual prevalence of the chronic conditions ranged from 1.0% to 24.7% (range 0-13),

For peer review only - http://bmjopen.bmj.com/site/about/guidelines.xhtml
For peer review only

with musculoskeletal disorder (24.7%) as the most prevalent chronic condition, followed by obesity (20.7%) and hypertension (18.4%). More than half of those with a chronic condition lived with multimorbidity. Additionally, depressive symptoms (not included in multimorbidity count) were observed to be affecting 17.6% of the study population. The prevalence of multimorbidity also increased steadily with age, from 9.6% among those younger than 35 years to as high as 46.8% in the elderly group (Table 2). The average number of chronic conditions tripled from the youngest adult group to the oldest age group. The prevalence of obesity and overweight was observed to be at an alarming stage, as was present in nearly 40% of the young adults (18–34 years), 62.8% of the middle-aged group (35–59 years), and over half (54.7%) of the elderly population (aged 60 and above) (Table 2).

Table 2

Clinical characteristics of the study cohort (overall and stratified by age group) (n=18,101)

	**Overall (n=18,101)**	**Age groups (years)**			***p*-value**
		**18–34 (n=4654) (25.7%)**	**35–59 (n=9029) (49.9%)**	**60 and above (n=4418) (24.4%)**	
Chronic conditions, n (%)					**< 0.001**
Heart disease	553 (3.2%)	46 (1.0%)	207 (2.4%)	300 (7.0%)	
Stroke	208 (1.2%)	7 (0.2%)	87 (1.0%)	114 (2.6%)	
Diabetes mellitus[†]	1868 (10.3%)	44 (0.9%)	975 (10.8%)	849 (19.2%)	
Hypertension[†]	3325 (18.4%)	75 (1.6%)	1633 (18.1%)	1617 (36.6%)	
Chronic kidney disease	173 (1.0%)	17 (0.4%)	68 (0.8%)	88 (2.0%)	
Arthritis, joint pain and/or back pain	4296 (24.7%)	509 (11.5%)	2033 (23.4%)	1754 (40.7%)	
Asthma	666 (3.8%)	123 (2.8%)	310 (3.5%)	233 (5.4%)	
Obesity	3665 (20.7%)	712 (15.4%)	2202 (24.7%)	751 (18.0%)	
Vision problem	2247 (12.4%)	221 (4.7%)	1182 (13.1%)	844 (19.1%)	
Hearing problem	489 (2.7%)	75 (1.6%)	154 (1.7%)	260 (5.9%)	
Physical mobility problem	1032 (5.7%)	238 (5.1%)	481 (5.3%)	313 (7.1%)	
Multimorbidity					
Mean number of conditions±SD	1.0±1.2	0.5±0.8	1.1±1.2	1.6±1.4	**< 0.001**
n (%)					
No multimorbidity	11,688 (71.5%)	3777 (90.4%)	5803 (70.6%)	2108 (53.2%)	**< 0.001**
Zero condition	7237 (44.3%)	2815 (67.4%)	3412 (41.5%)	1010 (25.5%)	
One condition	4449 (27.2%)	962 (23.0%)	2389 (29.1%)	1098 (27.7%)	
With multimorbidity	4669 (28.5%)	401 (9.6%)	2413 (29.4%)	1855 (46.8%)	

For peer review only

For peer review only - http://bmjopen.bmj.com/site/about/guidelines.xhtml

Two conditions	2595 (15.9%)	287 (6.9%)	1424 (17.3%)	884 (22.3%)	
Three conditions	1278 (7.8%)	92 (2.2%)	627 (7.6%)	559 (14.1%)	
≥ Four conditions	791 (4.8%)	21 (0.5%)	360 (4.4%)	410 (10.4%)	
BMI categories					**< 0.001**
Obesity (BMI ≥ 30.0 kg/m²)	3665 (20.7%)	712 (15.4%)	2202 (24.7%)	751 (18.0%)	
Overweight (BMI 25.0–29.9 kg/m²)	5994 (33.8%)	1063 (23.0%)	3396 (38.1%)	1535 (36.7%)	
Normal (BMI 18.5–24.9 kg/m²)	7309 (41.3%)	2452 (53.1%)	3132 (35.1%)	1725 (41.3%)	
Underweight (BMI < 18.5 kg/m²)	746 (4.2%)	390 (8.4%)	186 (2.1%)	170 (4.1%)	
Depressive symptoms, n (%)					**< 0.001**
Yes	3138 (17.6%)	891 (19.6%)	1570 (17.7%)	677 (15.6%)	
Medication prescription, n (%)					
Antidiabetic medication	1023/1537 (66.6%)	10 (23.8%)	531 (66.0%)	482 (69.9%)	**< 0.001**
Insulin	425/1537 (27.7%)	4 (9.5%)	232 (28.8%)	189 (27.4%)	**0.024**
Oral hypoglycaemic agents	920/1540 (59.7%)	8 (19.0%)	479 (59.4%)	433 (62.6%)	**< 0.001**
Antihypertensive therapy	1871/2847 (65.7%)	19 (27.9%)	857 (62.6%)	995 (70.6%)	**< 0.001**
Hospitalization (≥ 1 night in the past 6 months)¥, n (%)					NA
Yes	–	–	–	320/4414 (7.0%)	
Emergency Department visit (in the past 6 months)¥, n (%)					NA
Yes	–	–	–	324/4414 (4.8%)	

Abbreviations: SD=Standard deviation, BMI=Body mass index, NA=Not applicable
Numbers may not add up to totals due to missing data.
†Self-reported diagnosis or medication use.
¥Only participants aged 60 years and above.
Statistical significance testing by chi-square (χ^2) test and one-way ANOVA with post-hoc Bonferroni test.

Figure 2 displays the graphical trends of the number of chronic conditions and multimorbidity of the study population by age groups (18–24, 25–34, 35–44, 45–54, 55–64, ≥ 65 years). The number of chronic conditions and multimorbidity increased linearly with age ($p < 0.001$). The prevalence of any chronic condition increased from 27.9% in the youngest age group (18–24 years) to as high as 55.7% in the group of ≥ 65 years (Figure 2).

The results of multivariable logistic regression analysis assessing the association between socio-demographics and multimorbidity are shown in Table 3. Older age was associated with a higher likelihood of having multimorbidity (adjusted OR=1.05, 95% CI=1.05–1.05).

For peer review only

For peer review only - http://bmjopen.bmj.com/site/about/guidelines.xhtml

Table 3 Multivariable logistic regression modelling results of factors associated with multimorbidity (yes/no)

Parameter	Crude OR	Adjusted -OR	95% Confidence interval (CI)		*p* value
			Lower bound	Upper bound	
**Age**	1.05*	1.05	1.05	1.05	**< 0.001**
**Sex**					**< 0.001**
Male (Ref)		1			
Female	1.33*	1.28	1.16	1.40	
**Ethnicity**					0.244
Malay (Ref)		1			
Non-Malay	1.06	1.05	0.97	1.13	
**Education level**					**< 0.001**
No formal education (Ref)		1			
Primary	0.63*	0.64	0.54	0.75	**< 0.001**
Secondary	0.66*	0.60	0.51	0.71	**< 0.001**
Tertiary	0.72*	0.65	0.54	0.80	**< 0.001**
**Employment status**					**< 0.001**
Working/ Self-employed	0.30*	0.70	0.60	0.81	**< 0.001**
Student/Homemaker/ Unemployed	0.60*	0.87	0.74	1.02	0.081
Retiree (Ref)		1			

Abbreviation: Ref=Reference group, OR=Odds Ratio.
Diagnostic tests for complete case models: Nagelkerke R^2= 0.159.
*Significant *p* value for crude OR.

In addition to older age, results from the multivariable logistic regression show that multimorbidity was associated with other socio-demographic factors, such as female sex, education level, and employment status (Table 3). Specifically, women are 1.28 times (95%

CI=1.16–1.40) more likely to experience multimorbidity compared to men. Similarly, having no formal education increased the odds of developing multimorbidity (primary education: adjusted-OR=0.64, 95% CI=0.54–0.75, secondary education: adjusted-OR=0.60, 95% CI=0.51–0.71; tertiary education: adjusted-OR=0.65, 95% CI=0.54–0.80). Working group was less likely to develop multimorbidity than the retirees (adjusted-OR=0.70, 95% CI=0.60–0.81).

DISCUSSIONS

This population-based study provided evidence on the magnitude of multimorbidity and individual chronic conditions in a HDSS-scale community-dwelling general adult population. In addition, we identified various socio-demographic factors that could play a significant role in contributing to multimorbidity.

Prevalence of multimorbidity and individual chronic conditions

Using simple disease counts of 11 selected chronic conditions, we estimated the prevalence of multimorbidity at 28.5% in the overall study population. Musculoskeletal disorder, obesity, hypertension, and depressive symptoms are the most prominent physical and mental health conditions in this population. As expected, multimorbidity is more prevalent in older adults, affecting nearly half of the individuals aged 65 years and older (46.8%). However, it is worth noting that multimorbidity is affecting almost 10% of the young adults (18–34 years) and approximately one-third of the middle-aged adults (35–59 years), implying that multimorbidity is not just an issue of the elderly.

Extensive global and regional research has been conducted, showing substantial variability in the prevalence of multimorbidity. A systematic review and meta-analysis of 68

For peer review only - http://bmjopen.bmj.com/site/about/guidelines.xhtml

community-based studies worldwide by Nguyen and colleagues (2019) reported a prevalence range of multimorbidity of 3.5% to 70% in 37 HICs, and 1% to 90% in 31 LMICs (8). However, the number of diseases used as cut-off points for disease counts, disease combination, age ranges, and definitions of multimorbidity differ widely across the studies. These inevitable heterogeneities, subsequently, generated inconsistent measures and prevalence of multimorbidity that hindered the comparability of findings between studies. The prevalence of multimorbidity of our studies is similar but higher than the few local studies (12-15), including the only three studies conducted by in-country researchers (15-17). The measures of these studies were constrained by the number of considered chronic conditions (i.e. < 10 diseases), relatively old data (i.e. 20 years ago), or only involved older cohorts (aged ≥ 56 years). Therefore, the present study has addressed recent calls for investigations in general and multi-ethnic population samples, assessing a broader range of chronic conditions characterised by real-world community-based large data comprising medical diagnoses, anthropometric measurements, and environmental risk factors.

Factors associated with multimorbidity

Our findings are in agreement with the known paradox and other studies that multimorbidity increases with age, as long-term diseases accumulate with age (10, 23). It is interesting to note that in our study population, older age only explained part of the augmented risk of multimorbidity. We identified that other factors (sex, education level and employment status) are responsible for a substantial part of the association with multimorbidity. The inconsistency in sex differences and multimorbidity is not new, although most studies reported a higher prevalence of multimorbidity in women (24). In this study, women were also more likely to be affected by multimorbidity. The findings are congruent with the

For peer review only - http://bmjopen.bmj.com/site/about/guidelines.xhtml

literature in LMICs (15, 25), but not to the findings of Collaborative Research on Ageing in Europe (COURAGE) study conducted in Finland, Poland and Spain (24), the HICs. This discrepancy could be attributed to biological, social, and physical differences that generated inequalities in health status and life expectancy. Education level appears to be associated with a higher risk of multimorbidity in our study. We found that individuals in general with no education had a higher likelihood of having multimorbidity than their counterparts. This is broadly in line with numerous other studies (23, 25, 27, 28). The impact of education on multimorbidity might be due to higher socio-economic status and healthier behaviours and knowledge of individuals with higher education than those with less-educated backgrounds (28). Employment status was associated with multimorbidity in the study population. Compared to retirees, working adults had a lower likelihood of developing multimorbidity. Age factor may potentially be underlying this relationship; this is not impossible when retirees are generally older than the working individuals.

Strengths and limitations

The strengths of the present study include the use of population-based dataset with high participation and completion rate (71.1%) that includes individuals of a broad range of ages, including those where chronic disease load begins to manifest. We have also captured a more comprehensive range of current and chronic conditions of ageing that have not been included in previous studies of multimorbidity. To the best of our knowledge, this is the first study investigating multimorbidity in a HDSS-scale real-world community-based general cohort in Malaysia. The breath of sophisticated measures available for use as covariates, spanning socio-economic and demographic domains, is a further strength of the current study, as it

For peer review only

allowed a more thorough investigation of factors associated with multimorbidity than is usually attainable with medical records.

However, the current research is not without limitations. First, causality cannot be assessed based on the nature of chronic condition data and hence cross-sectional study design. Future longitudinal investigation would allow an opportunity for causal inference. Second, the clinical diagnoses of medical conditions were self-reported by the participants, thus, may be susceptible to bias and inaccuracies that deviate from medical records. Nevertheless, self-report of diagnosis of chronic conditions has been widely used in epidemiological studies and proven valid (33). Third, the reports of chronic conditions were restricted to the list included in the interview. As such, the prevalence of multimorbidity reported may be underestimated. However, we have selected the survey rounds with the longest lists of chronic conditions possible in our longitudinal databases and have included all relevant diseases for the assessment. Thus, we provided a detailed insight into multimorbidity in the study population. Lastly, it should be acknowledged that the surveys were conducted on participants who resided in a semi-rural area. In this regard, certain groups of the population may be under-represented, such as those with higher socio-economic status, and patients who utilised private healthcare facilities centred around urban areas with more optimal disease control.

Implications for policy, practice, and future research

The knowledge and evidence deficiencies of the epidemiology of multimorbidity in Malaysia are now addressed. They are vital steps towards future research and sensible governance and policy development to prevent developing multimorbidity and tackle the growing problems

For peer review only - http://bmjopen.bmj.com/site/about/guidelines.xhtml

For peer review only

multimorbidity brings. Our findings facilitate the identification of high-risk populations and suggest a scope for a prevention strategy focusing on reducing the development of multimorbidity. Given the high prevalence of multimorbidity and the expected increase in the future, the current single-disease-specific design of the health system should also be accompanied by strategies to address complexities associated with multimorbidity. Our findings suggest that the key to managing multimorbidity may be to strengthen a realistic and multidisciplinary approach that simultaneously targets health outcomes and socio-economic and demographics. The findings of this study could also be applicable for improving health outcomes and policy-making in other countries with social and economic developments comparable to that in Malaysia. We propose further research on the efficacy and effectiveness of trials are critical to be tested and established in better-equipped scientific settings with trained research staff. Therefore, funding and initiatives are important to translate the research findings into real-world settings, while exploring cost-effectiveness as a dual strategy in a resource-limited setting. Identifying specific clusters of chronic diseases that could cause the most significant problems is beyond the scope of this study, albeit its importance. Further research on multimorbidity clusters alongside their risk factors is needed to clarify relevant mechanisms to facilitate the development of targeted interventions for patients.

CONCLUSION

The study shows that multimorbidity is common among adults of any age. The prevalence and risk of multimorbidity increased with age. Multimorbidity is also associated with sex, education level, and employment status, indicating that multimorbidity is not just an issue of the elderly. Besides contributing to the developing literature for multimorbidity in LMICs, this

For peer review only

study highlights the importance of prioritising health policies, integrated care interventions, and clinical practice. All these should be oriented to implementing and delivering changes that include evidence-based components for effective preventative intervention and management of multimorbidity.

Contributors

Conceptualization: MMCT, MP, GMT, DM, TTS. Data Curation: MMCT, MRI. Formal Analysis: MMCT. Funding Acquisition: MP, GMT, DM, TTS. Investigation: MMCT, MP, GMT, PA, DR, TTS. Methodology: MMCT, MP, GMT, DM, PA, DR, TTS. Project Administration: MMCT. Resources: MMCT, MP, GMT, PA, DR, TTS. Software: MMCT, MP. Supervision: MMCT, MP, GMT, TTS. Visualization: MMCT. Writing – Original Draft Preparation: MMCT. Writing – Review & Editing: MMCT, MP, GMT, DM, EA, AÁMK, PA, DR.

Ethics Approvals

Ethics approvals were obtained from the Monash University Human Research Ethics Committee (MUHREC CF11/3663 – 2011001930 and 2013-3837-3646). All participants had provided written consent prior to data collection.

Funding

This work and publication of this article were supported by the Medical Research Council (UK Research and Innovation) grant (MUTUAL grant, King's College London, Grant number MR/T037423/1 awarded to MP).

Disclaimer

The funders had no role in study design and the execution of the study.

Data Availability

Data is available on request from the corresponding authors, or by application to the South East Asia Community Observatory (SEACO), Jeffrey Cheah School of Medicine and Health Sciences, Monash University Malaysia.

Competing Interests

The authors have declared that no competing interests exist.

Open Access

This is an Open Access article distributed in accordance with the Creative Commons Attribution (CC BY 4.0) license, which permits others to copy, redistribute, remix, transform and build upon this work for any purpose, provided the original work is properly cited, a link to the licence is given, and indication of whether changes were made. See: https://creativecommons.org/licenses/by/4.0/.

For peer review only - http://bmjopen.bmj.com/site/about/guidelines.xhtml

Figures

Figure 1 Study sampling flowchart

Figure 2 Graphical representation of the prevalence of multimorbidity and number of chronic conditions of the study population by age groups (n=18,101)

REFERENCES

1. World Health Organization (WHO). Multimorbidity: Technical series on safer primary care. Geneva: World Health Organization; 2016.
2. The Academy of Medical Sciences UK. Multimorbidity: A priority for global health research. Academy of Medical Sciences, London; 2018 April 2018.
3. Smith SM, Soubhi H, Fortin M, Hudon C, O'Dowd T. Managing patients with multimorbidity: systematic review of interventions in primary care and community settings. BMJ. 2012;345:e5205.
4. Fortin M, Lapointe L, Hudon C, Vanasse A, Ntetu AL, Maltais D. Multimorbidity and quality of life in primary care: A systematic review. Health and Quality of life Outcomes. 2004;2(1):1-12.
5. Lehnert T, Heider D, Leicht H, Heinrich S, Corrieri S, Luppa M, et al. Health care utilization and costs of elderly persons with multiple chronic conditions. Medical Care Research and Review. 2011;68(4):387-420.
6. Gallo JJ, Hwang S, Joo JH, Bogner HR, Morales KH, Bruce ML, et al. Multimorbidity, depression, and mortality in primary care: randomized clinical trial of an evidence-based depression care management program on mortality risk. Journal of General Internal Medicine. 2016;31(4):380-6.
7. Stirland LE, González-Saavedra L, Mullin DS, Ritchie CW, Muniz-Terrera G, Russ TC. Measuring multimorbidity beyond counting diseases: systematic review of community and population studies and guide to index choice. BMJ. 2020;368.
8. Nguyen H, Manolova G, Daskalopoulou C, Vitoratou S, Prince M, Prina AM. Prevalence of multimorbidity in community settings: A systematic review and meta-analysis of observational studies. Journal of Comorbidity. 2019;9:2235042X19870934.
9. van Oostrom SH, Gijsen R, Stirbu I, Korevaar JC, Schellevis FG, Picavet HSJ, et al. Time trends in prevalence of chronic diseases and multimorbidity not only due to aging: Data from general practices and health surveys. PlOS One. 2016;11(8):e0160264.
10. Jürisson M, Pisarev H, Uusküla A, Lang K, Oona M, Kalda R. Prevalence of chronic conditions and multimorbidity in Estonia: A population-based cross-sectional study. BMJ Open. 2021;11(10):e049045.
11. The World Bank IBRD IDA. World Bank Open Data 2021 [Available from: http://data.worldbank.org/.
12. Tey NP, Siraj SB, Kamaruzzaman SBB, Chin AV, Tan MP, Sinnappan GS, et al. Aging in multi-ethnic Malaysia. The Gerontologist. 2016;56(4):603-9.
13. Stubbs B, Vancampfort D, Veronese N, Kahl K, Mitchell A, Lin P-Y, et al. Depression and physical health multimorbidity: primary data and country-wide meta-analysis of population data from 190 593 people across 43 low-and middle-income countries. Psychological Medicine. 2017;47(12):2107-17.

For peer review only - http://bmjopen.bmj.com/site/about/guidelines.xhtml

14. Afshar S, Roderick PJ, Kowal P, Dimitrov BD, Hill AG. Global patterns of multimorbidity: a comparison of 28 countries using the world health surveys. Applied Demography and Public Health in the 21st Century: Springer; 2017. p. 381-402.
15. Hussin NM, Shahar S, Din NC, Singh DKA, Chin A-V, Razali R, et al. Incidence and predictors of multimorbidity among a multiethnic population in Malaysia: a community-based longitudinal study. Aging Clinical & Experimental Research. 2019;31(2):215-24.
16. Yap KH, Warren N, Allotey P, Reidpath DD. Chronic disease profiles of subjective memory complaints: a latent class analysis of older people in a rural Malaysian community. Aging & Mental Health. 2020;24(5):709-16.
17. Ghazali SS, Seman Z, Zainuddin NH, Omar MA, Sooryanarayana R, Ariaratnam S, et al. Prevalence and factors associated with multimorbidity among older adults in Malaysia: a population-based cross-sectional study. BMJ Open. 2021;11(10):e052126.
18. Partap U, Young EH, Allotey P, Soyiri IN, Jahan N, Komahan K, et al. HDSS profile: the South East Asia community observatory health and demographic surveillance system (SEACO HDSS). International Journal of Epidemiology. 2017;46(5):1370-1g.
19. World Health Organization (WHO). Body mass index - BMI Geneva: World Health Organization (WHO); 2019 [Available from: http://www.euro.who.int/en/health-topics/disease-prevention/nutrition/a-healthy-lifestyle/body-mass-index-bmi.
20. Henry JD, Crawford JR. The short-form version of the Depression Anxiety Stress Scales (DASS-21): Construct validity and normative data in a large non-clinical sample. British Journal of Clinical Psychology. 2005;44(2):227-39.
21. Lovibond PF, Lovibond SH. The structure of negative emotional states: Comparison of the Depression Anxiety Stress Scales (DASS) with the Beck Depression and Anxiety Inventories. Behaviour Research and Therapy. 1995;33(3):335-43.
22. Armstrong T, Bull F. Development of the world health organization global physical activity questionnaire (GPAQ). Journal of Public Health. 2006;14(2):66-70.
23. Blümel JE, Carrillo-Larco RM, Vallejo MS, Chedraui P. Multimorbidity in a cohort of middle-aged women: Risk factors and disease clustering. Maturitas. 2020;137:45-9.
24. Garin N, Koyanagi A, Chatterji S, Tyrovolas S, Olaya B, Leonardi M, et al. Global multimorbidity patterns: a cross-sectional, population-based, multi-country study. Journals of Gerontology Series A: Biomedical Sciences and Medical Sciences. 2016;71(2):205-14.
25. Jankovic J, Mirkovic M, Jovic-Vranes A, Santric-Milicevic M, Terzic-Supic Z. Association between non-communicable disease multimorbidity and health care utilization in a middle-income country: Population-based study. Public Health. 2018;155:35-42.
26. Ashworth M, Durbaba S, Whitney D, Crompton J, Wright M, Dodhia H. Journey to multimorbidity: longitudinal analysis exploring cardiovascular risk factors and sociodemographic determinants in an urban setting. BMJ Open. 2019;9(12):e031649.
27. Johnson-Lawrence V, Zajacova A, Sneed R. Education, race/ethnicity, and multimorbidity among adults aged 30–64 in the National Health Interview Survey. SSM-Population Health. 2017;3:366-72.
28. Demirchyan A, Khachadourian V, Armenian HK, Petrosyan V. Short and long term determinants of incident multimorbidity in a cohort of 1988 earthquake survivors in Armenia. International Journal for Equity in Health. 2013;12(1):1-8.
29. Mounce LT, Campbell JL, Henley WE, Arreal MCT, Porter I, Valderas JM. Predicting incident multimorbidity. The Annals of Family Medicine. 2018;16(4):322-9.

For peer review only - http://bmjopen.bmj.com/site/about/guidelines.xhtml

30. Blumental-Perry A. Unfolded protein response in chronic obstructive pulmonary disease: smoking, aging and disease: a SAD trifecta. Current Molecular Medicine. 2012;12(7):883-98.
31. Vancampfort D, Koyanagi A, Ward PB, Rosenbaum S, Schuch FB, Mugisha J, et al. Chronic physical conditions, multimorbidity and physical activity across 46 low-and middle-income countries. International Journal of Behavioral Nutrition and Physical Activity. 2017;14(1):1-13.
32. Vancampfort D, Stubbs B, Koyanagi A. Physical chronic conditions, multimorbidity and sedentary behavior amongst middle-aged and older adults in six low-and middle-income countries. International Journal of Behavioral Nutrition and Physical Activity. 2017;14(1):1-13.
33. Simpson CF, Boyd CM, Carlson MC, Griswold ME, Guralnik JM, Fried LP. Agreement between self-report of disease diagnoses and medical record validation in disabled older women: factors that modify agreement. Journal of the American Geriatrics Society. 2004;52(1):123-7.

For peer review only

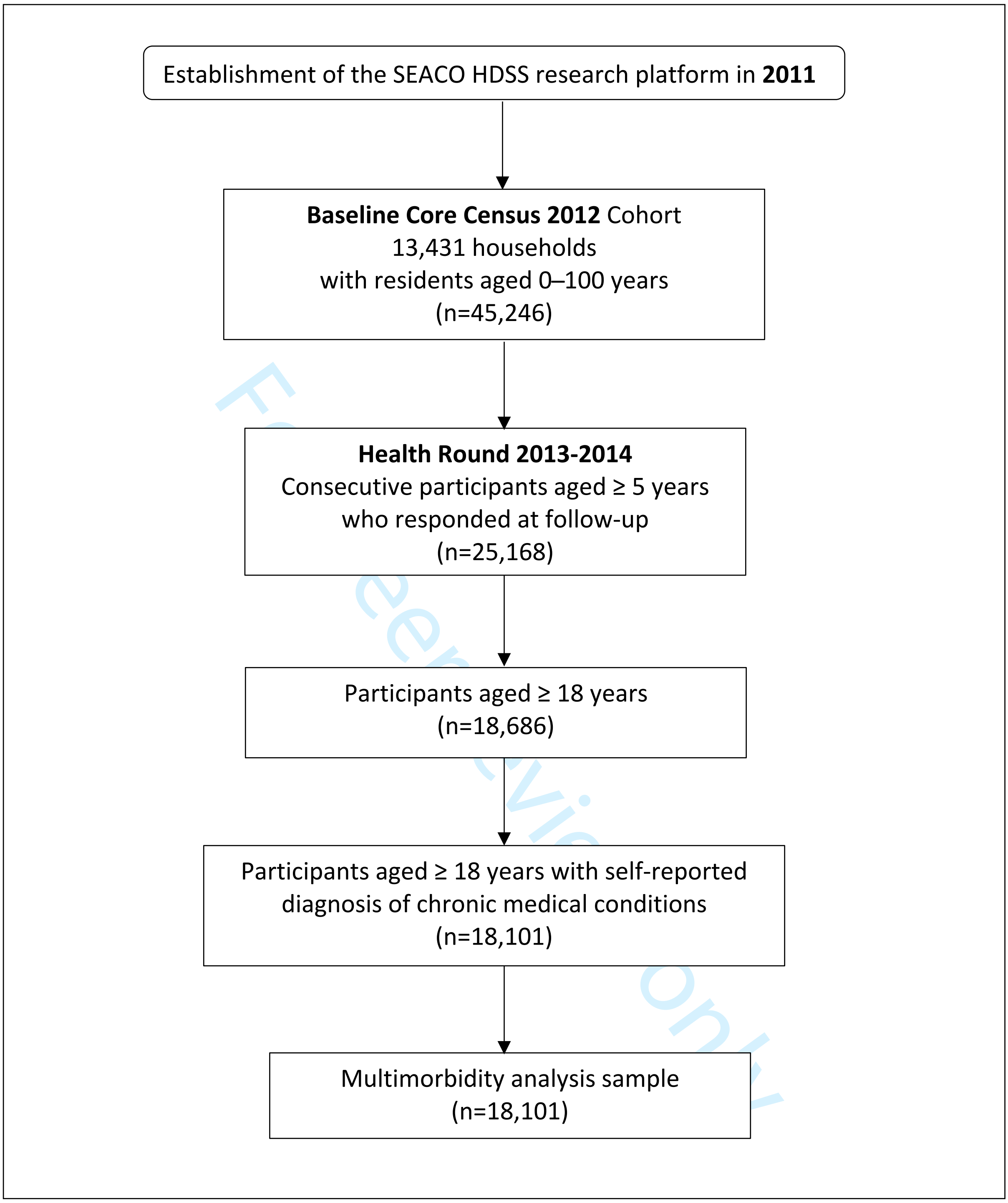

Figure 1 Study sampling flowchart

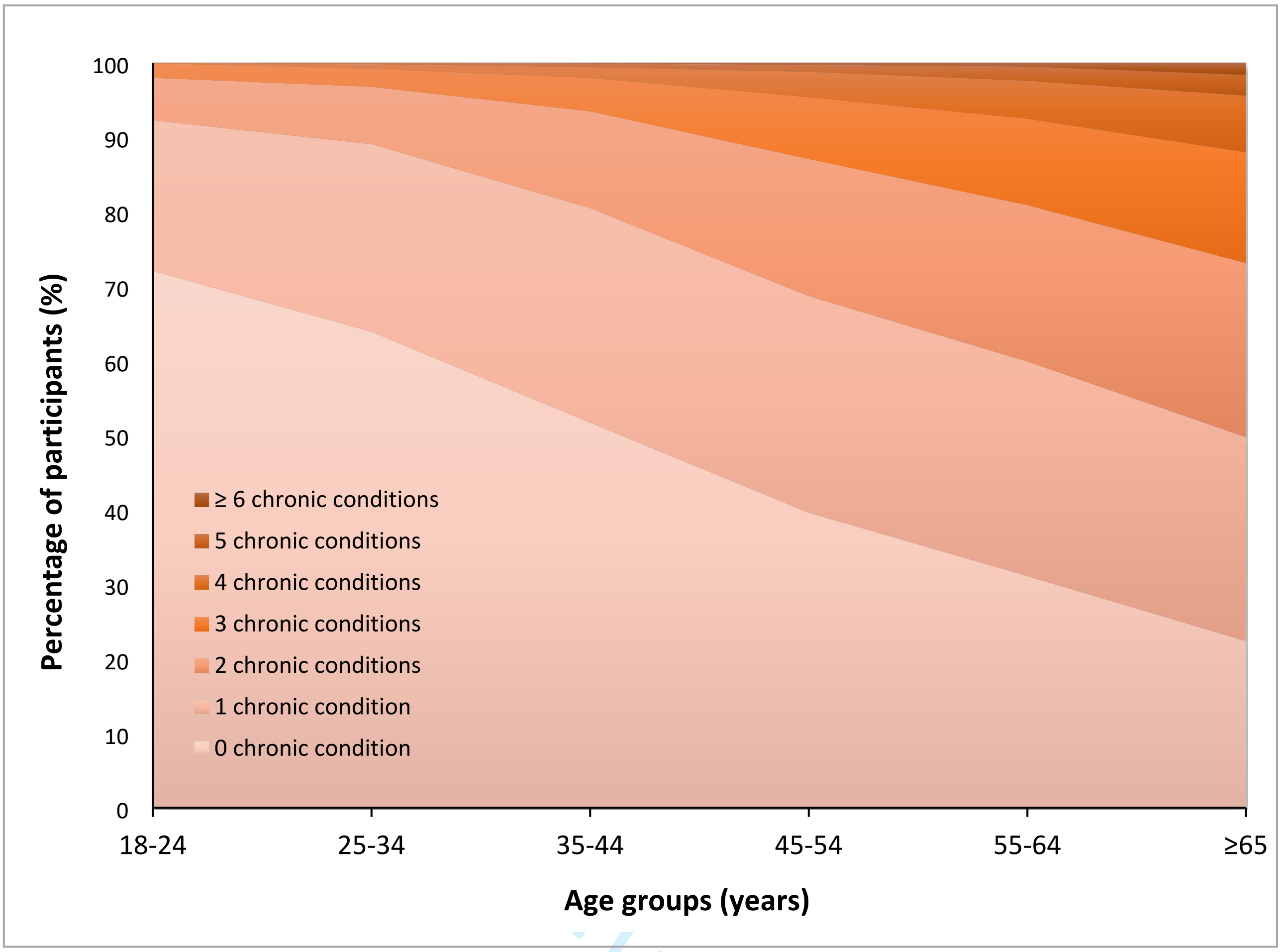

Figure 2 Graphical representation of the prevalence of multimorbidity and number of chronic conditions of the study population by age groups (n=18,101).

STROBE Statement—Checklist of items that should be included in reports of ***cross-sectional studies***

	Item No	Recommendation	Page No
**Title and abstract**	1	(*a*) Indicate the study's design with a commonly used term in the title or the abstract	1, 3
		(*b*) Provide in the abstract an informative and balanced summary of what was done and what was found	1, 3
**Introduction**			
Background/rationale	2	Explain the scientific background and rationale for the investigation being reported	3-5
Objectives	3	State specific objectives, including any prespecified hypotheses	5
**Methods**			
Study design	4	Present key elements of study design early in the paper	5
Setting	5	Describe the setting, locations, and relevant dates, including periods of recruitment, exposure, follow-up, and data collection	5, 6
Participants	6	(*a*) Give the eligibility criteria, and the sources and methods of selection of participants	6, 7
Variables	7	Clearly define all outcomes, exposures, predictors, potential confounders, and effect modifiers. Give diagnostic criteria, if applicable	8-10
Data sources/ measurement	8*	For each variable of interest, give sources of data and details of methods of assessment (measurement). Describe comparability of assessment methods if there is more than one group	8-10
Bias	9	Describe any efforts to address potential sources of bias	N/A
Study size	10	Explain how the study size was arrived at	7
Quantitative variables	11	Explain how quantitative variables were handled in the analyses. If applicable, describe which groupings were chosen and why	10
Statistical methods	12	(*a*) Describe all statistical methods, including those used to control for confounding	10
		(*b*) Describe any methods used to examine subgroups and interactions	10
		(*c*) Explain how missing data were addressed	N/A
		(*d*) If applicable, describe analytical methods taking account of sampling strategy	10
		(*e*) Describe any sensitivity analyses	N/A
**Results**			
Participants	13*	(a) Report numbers of individuals at each stage of study—eg numbers potentially eligible, examined for eligibility, confirmed eligible, included in the study, completing follow-up, and analysed	7, 10, 11
		(b) Give reasons for non-participation at each stage	7
		(c) Consider use of a flow diagram	7
Descriptive data	14*	(a) Give characteristics of study participants (eg demographic, clinical, social) and information on exposures and potential confounders	7-13
		(b) Indicate number of participants with missing data for each variable of interest	Footnotes of Tables 1 & 2

For peer review only - http://bmjopen.bmj.com/site/about/guidelines.xhtml

Outcome data	15*	Report numbers of outcome events or summary measures	14-16
Main results	16	(*a*) Give unadjusted estimates and, if applicable, confounder-adjusted estimates and their precision (eg, 95% confidence interval). Make clear which confounders were adjusted for and why they were included	15, 16
		(*b*) Report category boundaries when continuous variables were categorized	9, 10
		(*c*) If relevant, consider translating estimates of relative risk into absolute risk for a meaningful time period	N/A
Other analyses	17	Report other analyses done—eg analyses of subgroups and interactions, and sensitivity analyses	10
**Discussion**			
Key results	18	Summarise key results with reference to study objectives	16
Limitations	19	Discuss limitations of the study, taking into account sources of potential bias or imprecision. Discuss both direction and magnitude of any potential bias	18-20
Interpretation	20	Give a cautious overall interpretation of results considering objectives, limitations, multiplicity of analyses, results from similar studies, and other relevant evidence	16-20
Generalisability	21	Discuss the generalisability (external validity) of the study results	20-21
**Other information**			
Funding	22	Give the source of funding and the role of the funders for the present study and, if applicable, for the original study on which the present article is based	21

*Give information separately for exposed and unexposed groups.

Note: An Explanation and Elaboration article discusses each checklist item and gives methodological background and published examples of transparent reporting. The STROBE checklist is best used in conjunction with this article (freely available on the Web sites of PLoS Medicine at http://www.plosmedicine.org/, Annals of Internal Medicine at http://www.annals.org/, and Epidemiology at http://www.epidem.com/). Information on the STROBE Initiative is available at www.strobe-statement.org.

For peer review only

BMJ Open

Prevalence of, and factors associated with, multimorbidity among 18,101 adults in the South East Asia Community Observatory Health and Demographic Surveillance System in Malaysia: a population-based, cross-sectional study of the MUTUAL consortium

Journal:	*BMJ Open*
Manuscript ID	bmjopen-2022-068172.R1
Article Type:	Original research
Date Submitted by the Author:	25-Nov-2022
Complete List of Authors:	Tan, Michelle; King's College London Institute of Psychiatry Psychology and Neuroscience, Department of Health Service and Population Research; Monash University Faculty of Medicine Nursing and Health Sciences, Victorian Heart Institute<br>Prina, A.Matthew; King's College London Institute of Psychiatry Psychology and Neuroscience, Department of Health Service and Population Research,<br>Muniz Terrera, Graciela; University of Edinburgh and Western General Hospital, Edinburgh Dementia Prevention; Ohio University, Department of Social Medicine, Heritage College of Osteopathic Medicine,<br>Mohan, Devi; Monash University Malaysia, Global Public Health, Jeffrey Cheah School of Medicine and Health Sciences<br>Ismail, Roshidi; Monash University Malaysia, South East Asia Community Observatory (SEACO), Jeffrey Cheah School of Medicine and Health Sciences<br>Assefa, Esubalew; Addis Ababa University, Centre for Innovative Drug Development and Therapeutic Trials for Africa (CDT-Africa), College of Health Sciences, ; Jimma University, Department of Economics, College of Business and Economics,<br>Keinert, Ana; Universidade Federal de São Paulo, Departamento de Psiquiatria e Psicologia Médica, Escola Paulista de Medicina,<br>bin Kassim, Zaid; Ministry of Health Malaysia, District Health Office Segamat<br>Allotey, P; United Nations University International Institute for Global Health, IIGH; World Health Organization (WHO), Department of Sexual and Reproductive Health and Research<br>Reidpath, Daniel; Monash University Malaysia, Jeffrey Cheah School of Medicine and Health Sciences; Queen Margaret University, Institute for Global Health and Development<br>Su, Tin ; Monash University - Malaysia Campus, Global Public Health, Jeffrey Cheah School of Medicine and Health Sciences; Monash University Malaysia, South East Asia Community Observatory (SEACO), Jeffrey Cheah School of Medicine and Health Sciences
**Primary Subject Heading**:	Epidemiology

Secondary Subject Heading:	Global health, Health policy, Health services research, Mental health, Public health
Keywords:	EPIDEMIOLOGY, HEALTH SERVICES ADMINISTRATION & MANAGEMENT, PUBLIC HEALTH

SCHOLARONE™
Manuscripts

I, the Submitting Author has the right to grant and does grant on behalf of all authors of the Work (as defined in the below author licence), an exclusive licence and/or a non-exclusive licence for contributions from authors who are: i) UK Crown employees; ii) where BMJ has agreed a CC-BY licence shall apply, and/or iii) in accordance with the terms applicable for US Federal Government officers or employees acting as part of their official duties; on a worldwide, perpetual, irrevocable, royalty-free basis to BMJ Publishing Group Ltd ("BMJ") its licensees and where the relevant Journal is co-owned by BMJ to the co-owners of the Journal, to publish the Work in this journal and any other BMJ products and to exploit all rights, as set out in our licence.

The Submitting Author accepts and understands that any supply made under these terms is made by BMJ to the Submitting Author unless you are acting as an employee on behalf of your employer or a postgraduate student of an affiliated institution which is paying any applicable article publishing charge ("APC") for Open Access articles. Where the Submitting Author wishes to make the Work available on an Open Access basis (and intends to pay the relevant APC), the terms of reuse of such Open Access shall be governed by a Creative Commons licence – details of these licences and which Creative Commons licence will apply to this Work are set out in our licence referred to above.

Other than as permitted in any relevant BMJ Author's Self Archiving Policies, I confirm this Work has not been accepted for publication elsewhere, is not being considered for publication elsewhere and does not duplicate material already published. I confirm all authors consent to publication of this Work and authorise the granting of this licence.

For peer review only

Prevalence of, and factors associated with, multimorbidity among 18,101 adults in the South East Asia Community Observatory Health and Demographic Surveillance System in Malaysia: a population-based, cross-sectional study of the MUTUAL consortium

Michelle M C Tan[1,2,3,4], Matthew Prina[1], Graciela Muniz-Terrera[5,6], Devi Mohan[2], Mohammad Roshidi Ismail[3], Esubalew Assefa[7,8,9], Ana Á M Keinert[10], Zaid Kassim[11], Pascale Allotey[2,12,13], Daniel Reidpath[3,14], and Tin Tin Su[2,3,4]

Author affiliations:

[1] Department of Health Service and Population Research, Institute of Psychiatry, Psychology & Neuroscience, King's College London, London, United Kingdom.
[2] Global Public Health, Jeffrey Cheah School of Medicine and Health Sciences, Monash University Malaysia, Sunway City, Selangor, Malaysia.
[3] South East Asia Community Observatory (SEACO), Jeffrey Cheah School of Medicine and Health Sciences, Monash University Malaysia, Sunway City, Selangor, Malaysia.
[4] Victorian Heart Institute, Clayton Campus, Faculty of Medicine, Nursing and Health Sciences, Monash University, Victoria, Australia.
[5] Edinburgh Dementia Prevention, University of Edinburgh and Western General Hospital, Edinburgh, Scotland, United Kingdom.
[6] Department of Social Medicine, Heritage College of Osteopathic Medicine, Ohio University, Ohio, United States.
[7] Centre for Innovative Drug Development and Therapeutic Trials for Africa (CDT-Africa), College of Health Sciences, Addis Ababa University, Addis Ababa, Ethiopia.
[8] Department of Economics, College of Business and Economics, Jimma University, Jimma, Ethiopia.
[9] Department of Economics, Faculty of Arts and Social Sciences, The Open University, Milton Keynes, United Kingdom.
[10] Departamento de Psiquiatria e Psicologia Médica, Escola Paulista de Medicina, Universidade Federal de São Paulo (UNIFESP), Rua Major Maragliano, Vila Mariana, São Paulo - SP, Brazil.
[11] District Health Office Segamat, Ministry of Health Malaysia, Johor, Malaysia.
[12] International Institute for Global Health, United Nations University, Kuala Lumpur, Malaysia.
[13] Department of Sexual and Reproductive Health and Research, World Health Organization (WHO), Geneva, Switzerland.
[14] Institute for Global Health and Development, Queen Margaret University, Edinburgh, Scotland, United Kingdom.

***Correspondence to:**

Dr Michelle M C Tan, PhD
tan_mun.chieng@kcl.ac.uk

Dr Matthew Prina, PhD
matthew.prina@kcl.ac.uk

Prof Tin Tin Su, PhD

For peer review only - http://bmjopen.bmj.com/site/about/guidelines.xhtml

TinTin.Su@monash.edu

ABSTRACT

Objectives To assess the prevalence and factors associated with multimorbidity in a community-dwelling general adult population on a large health and demographic surveillance system (HDSS) scale.

Design Population-based cross-sectional study.

Setting South East Asia Community Observatory (SEACO) HDSS site in Malaysia.

Participants Of 45,246 participants recruited from 13,431 households, 18,101 eligible adults aged 18–97 years (mean age 47 years, 55.6% female) were included.

Main outcome measures The main outcome was prevalence of multimorbidity. Multimorbidity was defined as the coexistence of two or more chronic conditions per individual. A total of 13 chronic diseases were selected and were further classified into 11 medical conditions to account for multimorbidity. The conditions were heart disease, stroke, diabetes mellitus, hypertension, chronic kidney disease, musculoskeletal disorder, obesity, asthma, vision problem, hearing problem, and physical mobility problem. Risk factors for multimorbidity were also analysed.

Results Of the study cohort, 28.5% people lived with multimorbidity. The individual prevalence of the chronic conditions ranged from 1.0% to 24.7%, with musculoskeletal disorder (24.7%), obesity (20.7%) and hypertension (18.4%) as the most prevalent chronic conditions. The number of chronic conditions increased linearly with age (p<0.001). In the logistic regression model, multimorbidity is associated with female sex (adjusted-OR=1.28, 95% CI=1.17–1.40, p<0.001), education levels (primary education compared to no education: adjusted-OR=0.63, 95% CI=0.53–0.74; secondary education: adjusted-OR=0.60, 95% CI=0.51–0.70; tertiary education: adjusted-OR=0.65, 95% CI=0.54–0.80; p<0.001), and employment status (working adults compared to retirees: adjusted-OR=0.70, 95% CI=0.60–0.82, p<0.001), in addition to age (adjusted-OR=1.05, 95% CI=1.05–1.05, p<0.001).

Conclusions The current single-disease services in primary and secondary care should be accompanied by strategies to address complexities associated with multimorbidity, taking into account the factors associated with multimorbidity identified. Future research is needed to identify the most commonly occurring clusters of chronic diseases and their risk factors to develop more efficient and effective multimorbidity prevention and treatment strategies.

Keywords: Multimorbidity; epidemiology; prevalence; risk factors; low- and middle-income countries.

STRENGTHS AND LIMITATIONS OF THIS STUDY

- This study examined the epidemiology of multimorbidity among community-dwelling adults on a large health and demographic surveillance system scale in a multi-ethnic and rapidly ageing middle-income country.
- Strengths of the study include the use of a population-based dataset with high participation and completion rate that includes individuals of a broad range of ages, including those where chronic disease load begins to manifest.

For peer review only - http://bmjopen.bmj.com/site/about/guidelines.xhtml

- We have also captured a more comprehensive range of current and chronic conditions of ageing that have not been included in previous studies of multimorbidity.
- Causality cannot be established due to the cross-sectional nature of the chronic condition data.

INTRODUCTION

Multimorbidity, as defined by the World Health Organization (WHO) (1), refers to the coexistence of two or more chronic health conditions in a single individual. The rising tide of multimorbidity is a key challenge for both patients and healthcare systems worldwide (2). Multimorbidity is associated with lower quality of life (3), impaired functional capacity (4), psychological distress (5), increased premature mortality (5), and greater healthcare service utilisation and medical expenditure (6).

There are numerous ways to measure multimorbidity, such as disease counts, conditions weighted or combined with other parameters, diagnostic categories, drug use, and physiological measures (7). Despite these various methods, a simple count of chronic diseases is the most popular method of measuring multimorbidity due to its ease of applying. In a recent systematic review and meta-analysis (8), it was found that the pooled prevalence of multimorbidity is comparable between high-income countries (HICs) (standardised-prevalence=37.9%, 95% CI=32.5–43.4%; non-standardised prevalence=41.3%, 95% CI=35.2–47.4%) and low- and middle-income countries (LMICs) (standardised-prevalence=29.7%, 95% CI=26.4–33.0%; non-standardised prevalence=43.5%, 95% CI=38.4–48.6%). While multimorbidity affects all nations and age groups, most studies of the prevalence of multimorbidity have focused on older populations in HICs, and data from younger adults, LMICs and those residing in socio-economically disadvantaged areas are notably lacking (2). Similarly, factors that may increase the risk of multimorbidity are poorly understood, with

For peer review only - http://bmjopen.bmj.com/site/about/guidelines.xhtml

ageing not being the only factor associated with high levels of multimorbidity (9). Consequently, it has not been possible to evaluate and develop patient-centred prevention and treatment strategies targeting multimorbidity and such risk factors. The COVID-19 pandemic further drew attention to the crucial contribution of multimorbidity to the need for sound public health measures and rapid identification of effective medical interventions grounded on risk stratification (10). To better manage the consequences of such an adverse phenomenon, a better understanding of the prevalence and factors associated with multimorbidity among diverse study populations became essential.

According to the World Bank Classification (11), Malaysia is a high-middle-income country in Asia, with a diverse population. After neighbouring Singapore, Malaysia is the second most urbanized country in South East Asia (12). While it is also experiencing a rapid demographic transition, edging towards population ageing and increasing prevalence of non-communicable diseases (12), there is limited information on multimorbidity of the general population in the real-world community setting. Very few studies have been able to evaluate the epidemiology of multimorbidity in Malaysia – the World Health Survey (13, 14) derived from multimorbidity measures that included a restricted number of conditions and relied on relatively old data. The only contextually-relevant studies by in-country researchers were either smaller in sample size (15) or included a limited number of chronic conditions (four and seven conditions, respectively) in the older population (16, 17).

This study is a response of the MUltimorbidity ThroUgh cApacity buiLding (MUTUAL) consortium, developed to better understand and address the growing challenge of multimorbidity in LMICs. The aims of this study were, therefore, to fill the evidence gaps by identifying the country-specific prevalence and factors associated with multimorbidity. To the best of our knowledge, this was the first study that examined in detail a community-dwelling

For peer review only

For peer review only - http://bmjopen.bmj.com/site/about/guidelines.xhtml

general adult population on a large health and demographic surveillance system (HDSS) scale in Malaysia. To underline the complexity of multimorbidity, and to understand the epidemiology in different age groups, we considered a comprehensive range of chronic health conditions and did not restrict our study to the elderly.

METHODS

Data source and study population

Data for the snapshot analyses were drawn from the dynamic South East Asia Community Observatory (SEACO) HDSS database established in 2011 in semi-urban and rural areas within the Segamat district of Johor state, Malaysia. The SEACO HDSS profile has been published previously (18), and is updated here. SEACO HDSS site has been capturing detailed longitudinal information related to health and diseases among individuals and households to improve the holistic experience in the local community and the wider population. SEACO remains the sole HDSS with International Standards Organization (ISO) certification for quality data collection and data management processes (ISO 9001:2015) among over 48 HDSS from LMICs worldwide (http://www.indepth-network.org/). Besides the MUTUAL consortium, SEACO is also a member of the International Network for the Demographic Evaluation of Populations and Their Health (INDEPTH) network and the recently-established HDSS-Asia network.

Comprehensive accounts of socio-demographic, lifestyle and medical information were gathered from all participants recruited to the population-wide baseline Core Census 2012 in an electronic handheld device-assisted face-to-face interview administered by trained interviewers. There were 45,246 community-dwelling residents aged 0–100 from 13,431 households enrolled in the Core Census 2012 for baseline enumeration. Of the participants,

For peer review only - http://bmjopen.bmj.com/site/about/guidelines.xhtml

38,228 participants were ≥ 18 years old (n=31,595) with a mean age of 45 years (SD 16.9 years). In 2013-2014, the first five-yearly population-wide health survey, Health Round Wave 1, was undertaken at the individual level among 25,168 participants aged 5 years and above who were part of the core sample. An overview of the population's overall health was assessed, including medical history, mental health, well-being, quality of life, lifestyle factors, and health service utilisation. Of them, 18,101 adults were aged 18 and older [mean age 47.3 years (SD 16.3 years)]. Data from this cohort of Health Round Wave 1 (2013/2014) and the Core Census 2012 were included in the current study for a longer list of chronic conditions to be considered. A flowchart of the population in this study is presented in Figure 1. Ethics approvals were obtained from the Monash University Human Research Ethics Committee (MUHREC CF11/3663 – 2011001930 and 2013-3837-3646). All participants had provided written consent prior to data collection.

Chronic condition measures and definition of multimorbidity

While chronic health conditions were self-reported and not directly based on formal clinical diagnoses, participants were asked whether they had ever been told by medical professionals that they had a particular chronic health condition based on a series of dichotomous questions (yes/no). The conditions were heart disease (including coronary artery disease, myocardial infarction, angina pectoris, congestive heart failure), stroke, type 1 and/or type 2 diabetes mellitus (DM), hypertension, chronic kidney disease, end-stage renal failure (ESRF) requiring dialysis, arthritis, joint pain, back pain, asthma, vision problem, hearing problem, and physical mobility problem.

Additionally, obesity (BMI ≥ 30 kg/m^2) was included and defined using the principal body mass index (BMI) classification by the WHO (19), by which weight (in kilograms) was

For peer review only - http://bmjopen.bmj.com/site/about/guidelines.xhtml

divided by height squared (in metres). Weight and height were estimated using electronic portable weighing scales with electronic height sensors (Patient Focus, model GBS-721), in which participant was barefoot and wearing light clothing during the measurement. Considering the heterogeneity in the multimorbidity definitions is always high, and obesity was not consistently included in the list of chronic conditions for multimorbidity despite being a chronic and complex condition, these may potentially underestimate the prevalence of multimorbidity. Moreover, including obesity in multimorbidity estimates is crucial due to the well-studied connections between obesity and a range of complications, including three out of the ten leading causes of death worldwide – ischaemic heart disease, stroke and type 2 diabetes mellitus (20). As such, we included obesity in the multimorbidity definition in our study.

Due to collinearity, participants who had any musculoskeletal disorder, either arthritis or joint pain or back pain or all three, were combined into one condition – 'arthritis, joint pain and/or back pain'. Likewise, the mutually exclusive chronic kidney disease and ESRF requiring dialysis were considered together as 'chronic kidney disease'. To avoid potential bias caused by spurious associations, medical conditions with a prevalence of ≤ 1% were excluded, such as speech problem and gastrointestinal conditions. Acute infectious diseases consisting of dengue fever and urinary tract infection were also excluded. Each of the included 11 chronic conditions was coded as binary, where presence=1 and absence=0. In the present study, multimorbidity is defined as the presence of two or more of these concurrent long-term physical conditions for each participant.

Mental health measures

For peer review only - http://bmjopen.bmj.com/site/about/guidelines.xhtml

Mental health was measured using the Depression, Anxiety and Stress Scale–21 (DASS-21) (21), the short form of the DASS-42 (22). In the SEACO HDSS site, the Malay version of DASS-21 previously translated and validated in Malaysia (23) was adopted. It is a set of three self-report scales designed to measure the emotional states of depression, anxiety, and stress. Each of the three DASS-21 scales contains 7 items that are divided into subscales with similar content. The subscales correspond to the following diagnostic categories: 'Depression' for mood disorders, 'Anxiety' for panic disorder and 'Stress' for generalized anxiety disorder. The individual scale items were scored on a 4-point Likert scale (never, sometimes, often, and almost always) of frequency or severity of participants' experiences over the past week. The score of each subscale was summed and multiplied by two to be comparable to the long form of DASS. Higher scores indicate greater levels of symptoms. Participants with a cut-off score of ≥10 were classified as having depressive symptoms (22).

Study variables

Socio-demographic variables such as age (18–24, 25–34, 35–44, 45–54, 55–64, ≥ 65), sex (female or male), ethnicity (Malay, Chinese, Indian, other), education attainment (no formal education, primary, secondary, tertiary education), employment status (working/self-employed, homemaker, unemployed, retired/pension, student), personal monthly gross income (< MYR 1,000, MYR 1,000-1,999, ≥ MYR 2,000), monthly household income [grouped into bottom 40% (B40), middle 40% (M40), top 20% (T20)], and marital status (categorised as married, never married, widowed/divorced/separated) were included. Private health insurance was coded into two categories (yes through self-subscription/employer, or no), and payer for health cost referred to either self/family member, government/pension, employer-provided health cover or personal health insurance. Health service utilisation included

For peer review only - http://bmjopen.bmj.com/site/about/guidelines.xhtml

medication prescriptions in the last two weeks (i.e., antidiabetic and antihypertensive drugs, coded into yes or no), hospital admissions (≥ 1 night in the past 6 months, categorised as yes or no) and emergency department visits in the past 6 months (yes or no).

Data analysis

Descriptive statistics were used to summarise information regarding socio-demographic variables, clinical profiles, health service utilisation, and the prevalence of multimorbidity and individual chronic diseases. The age groups were compared using one-way ANOVA with post-hoc Bonferroni test or chi-square (X^2) test. The multimorbidity groups (yes or no) were compared using independent *t*-test or X^2 test. The total number of chronic conditions was counted and categorised as no multimorbidity (0-1) and multimorbidity (2, 3 and ≥ 4 chronic conditions, respectively). A multivariable logistic regression model using the enter method with entry criteria of α=0.05 and removal criteria=0.10 was performed to identify factors associated with multimorbidity (yes/no), controlled for mental health (depressive symptoms) and socio-demographic covariates (age, sex, ethnicity, education level, and employment status). Covariates were selected, further categorised and included based on previous literature and theory. Diagnostic tests for complete case models were conducted. All analyses were performed using Stata/MP (Version 17, Stata Corp LP, College Station, TX, USA) and IBM SPSS Statistics (Version 28, Armonk, New York, USA). All statistical analyses were two-sided with a significance of $p<0.05$.

Patient and public involvement

None.

For peer review only - http://bmjopen.bmj.com/site/about/guidelines.xhtml

251 **RESULTS**

252 Socio-economic and demographic characteristics of the study cohort are reported in Table 1. 253 The average (range) age of the participants was 47 (18–97) years, 55.6% were females, two-254 thirds were Malays, and the majority were married. The study population largely drawn from 255 a semi-rural state in Malaysia was mainly represented by subjects who had lower education, 256 and household and personal income levels (Table 1).

257

258 **Table 1. Summary of population characteristics, showing socio-demographic**
259 **characteristics (overall and by multimorbidity status; n=18,101)**

	**Overall (n=18,101)**	**Multimorbidity**		***p*-value**
		**Yes (n=4669) (28.5%)**	**No (n=11,688) (71.5%)**	
Age (years)				**<0.001**
Mean±SD	47.3±16.3	55.3±13.8	44.0±15.8	
Sex, n (%)				**<0.001**
Female	9089 (55.6%)	2824 (60.5%)	6265 (53.6%)	
Male	7268 (44.4%)	1845 (39.5%)	5423 (46.4%)	
Ethnicity, n (%)				**0.005**
Malay	10,741 (65.7%)	3025 (64.9%)	7716 (66.1%)	
Chinese	3586 (21.9%)	1100 (23.6%)	2486 (21.3%)	
Indian	1594 (9.8%)	419 (9.0%)	1175 (10.1%)	
Other	418 (2.6%)	119 (2.6%)	299 (2.6%)	
Marital status, n (%)				**<0.001**
Never married	2584 (16.0%)	304 (6.5%)	2280 (19.8%)	
Married	11,846 (73.2%)	3568 (76.7%)	8278 (71.8%)	
Widowed/Divorced/Separated	1745 (10.8%)	781 (16.8%)	964 (8.4%)	
Education level, n (%)				**<0.001**
No formal education	841 (5.2%)	314 (6.9%)	527 (4.6%)	
Primary	4842 (30.1%)	1324 (28.9%)	3518 (30.6%)	
Secondary	9012 (56.1%)	2529 (55.2%)	6483 (56.4%)	
Tertiary	1373 (8.5%)	411 (9.0%)	962 (8.4%)	
Employment status, n (%)				**<0.001**
Working/Self-employed	8189 (50.3%)	1749 (37.6%)	6440 (55.4%)	
Homemaker	5017 (30.8%)	1704 (36.7%)	3313 (28.5%)	
Unemployed	1868 (11.5%)	743 (16.0%)	1125 (9.7%)	
Retired/Pension	885 (5.4%)	421 (9.1%)	464 (4.0%)	
Student	313 (1.9%)	32 (0.7%)	281 (2.4%)	
Personal gross monthly income (MYR)				**<0.001**
Mean±SD	1204.0±1515.4	1094.7±1684.1	1224.0±1383.4	
< 1,000	8554 (52.3%)	2707 (58.0%)	5847 (50.0%)	
1,000-1,999	4575 (28.0%)	1107 (23.7%)	3468 (29.7%)	

For peer review only - http://bmjopen.bmj.com/site/about/guidelines.xhtml

≥ 2,000	3228 (19.7%)	855 (18.3%)	2373 (20.3%)	
Household monthly income (MYR)†				**<0.001**
Mean±SD	3193.8±3537.1	2720.9±3333.0	3224.2±3357.7	
Bottom 40% (B40)	12,263 (75.0%)	3679 (78.8%)	8584 (73.4%)	
Middle 40% (M40)	3076 (18.8%)	749 (16.0%)	2327 (19.9%)	
Top 20% (T20)	1018 (6.2%)	241 (5.2%)	777 (6.6%)	
Private health insurance (Through employer/self-subscription), n (%)				**<0.001**
Yes	3650 (22.6%)	955 (20.7%)	2695 (23.3%)	
No	12,507 (77.4%)	3652 (79.3%)	8855 (76.7%)	
Payer for healthcare cost, n (%)				**<0.001**
Self/Family member	13,329 (82.0%)	3715 (80.1%)	9614 (82.8%)	
Government/Pension	2293 (14.1%)	805 (17.4%)	1488 (12.8%)	
Employer-provided health cover	492 (3.0%)	85 (1.8%)	407 (3.5%)	
Personal private health insurance	139 (0.9%)	32 (0.7%)	107 (0.9%)	

Abbreviations: SD=Standard deviation.
Numbers may not add up to totals due to missing data.
MYR=Malaysian Ringgit, MYR 1 was equivalent to the British Pound (GBP) £ 0.18 at the time of publication.
†Categorised as three income thresholds according to the local Department of Statistics, where B40, M40 and T20 represent groups with monthly income of < MYR 3860, MYR 3860–8319, and > MYR 8319, respectively.
Statistical significance testing by chi-square (χ^2) test and independent t-test.

Multimorbidity was identified in 28.5% of participants, with 15.9% having two conditions, and 12.6% having three or more conditions. See Table 2 for the clinical characteristics of the study cohort, including the prevalence of multimorbidity and individual chronic conditions. Approximately 44.3% of the cohort had no chronic condition, and 27.2% had one condition. The individual prevalence of the chronic conditions ranged from 1.0% to 24.7% (range 0-13), with musculoskeletal disorder (24.7%) as the most prevalent chronic condition, followed by obesity (20.7%) and hypertension (18.4%). More than half of those with a chronic condition lived with multimorbidity. Additionally, depressive symptoms (not included in multimorbidity count) were observed to be affecting 17.6% of the study population. The prevalence of multimorbidity also increased steadily with age, from 9.6% among those younger than 35 years to as high as 46.8% in the elderly group (Table 2). The average number of chronic conditions tripled from the youngest adult group to the oldest age group. The prevalence of obesity and overweight was observed to be at an alarming stage, as

For peer review only

was present in nearly 40% of the young adults (18–34 years), 62.8% of the middle-aged group (35–59 years), and over half (54.7%) of the elderly population (aged 60 and above) (Table 2).

Table 2. Clinical characteristics of the study cohort (overall and stratified by age group; n=18,101)

	Overall (n=18,101)	Age groups (years)			*p*-value
		18–34 (n=4654) (25.7%)	35–59 (n=9029) (49.9%)	60 and above (n=4418) (24.4%)	
Chronic conditions, n (%)					**<0.001**
Heart disease	553 (3.2%)	46 (1.0%)	207 (2.4%)	300 (7.0%)	
Stroke	208 (1.2%)	7 (0.2%)	87 (1.0%)	114 (2.6%)	
Diabetes mellitus†	1868 (10.3%)	44 (0.9%)	975 (10.8%)	849 (19.2%)	
Hypertension†	3325 (18.4%)	75 (1.6%)	1633 (18.1%)	1617 (36.6%)	
Chronic kidney disease	173 (1.0%)	17 (0.4%)	68 (0.8%)	88 (2.0%)	
Arthritis, joint pain and/or back pain	4296 (24.7%)	509 (11.5%)	2033 (23.4%)	1754 (40.7%)	
Asthma	666 (3.8%)	123 (2.8%)	310 (3.5%)	233 (5.4%)	
Obesity	3665 (20.7%)	712 (15.4%)	2202 (24.7%)	751 (18.0%)	
Vision problem	2247 (12.4%)	221 (4.7%)	1182 (13.1%)	844 (19.1%)	
Hearing problem	489 (2.7%)	75 (1.6%)	154 (1.7%)	260 (5.9%)	
Physical mobility problem	1032 (5.7%)	238 (5.1%)	481 (5.3%)	313 (7.1%)	
Multimorbidity					
Mean number of conditions±SD	1.0±1.2	0.5±0.8	1.1±1.2	1.6±1.4	**<0.001**
n (%)					
No multimorbidity	11,688 (71.5%)	3777 (90.4%)	5803 (70.6%)	2108 (53.2%)	**<0.001**
Zero condition	7237 (44.3%)	2815 (67.4%)	3412 (41.5%)	1010 (25.5%)	
One condition	4449 (27.2%)	962 (23.0%)	2389 (29.1%)	1098 (27.7%)	
With multimorbidity	4669 (28.5%)	401 (9.6%)	2413 (29.4%)	1855 (46.8%)	
Two conditions	2595 (15.9%)	287 (6.9%)	1424 (17.3%)	884 (22.3%)	
Three conditions	1278 (7.8%)	92 (2.2%)	627 (7.6%)	559 (14.1%)	
≥ Four conditions	791 (4.8%)	21 (0.5%)	360 (4.4%)	410 (10.4%)	
BMI categories					**<0.001**
Obesity (BMI ≥ 30.0 kg/m²)	3665 (20.7%)	712 (15.4%)	2202 (24.7%)	751 (18.0%)	
Overweight (BMI 25.0–29.9 kg/m²)	5994 (33.8%)	1063 (23.0%)	3396 (38.1%)	1535 (36.7%)	
Normal (BMI 18.5–24.9 kg/m²)	7309 (41.3%)	2452 (53.1%)	3132 (35.1%)	1725 (41.3%)	
Underweight (BMI < 18.5 kg/m²)	746 (4.2%)	390 (8.4%)	186 (2.1%)	170 (4.1%)	
Depressive symptoms, n (%)					**<0.001**
Yes	3138 (17.6%)	891 (19.6%)	1570 (17.7%)	677 (15.6%)	
Medication prescription, n (%)					
Antidiabetic medication	1023/1537 (66.6%)	10 (23.8%)	531 (66.0%)	482 (69.9%)	**<0.001**
Insulin	425/1537 (27.7%)	4 (9.5%)	232 (28.8%)	189 (27.4%)	**0.024**
Oral hypoglycaemic agents	920/1540 (59.7%)	8 (19.0%)	479 (59.4%)	433 (62.6%)	**<0.001**
Antihypertensive therapy	1871/2847 (65.7%)	19 (27.9%)	857 (62.6%)	995 (70.6%)	**<0.001**
Hospitalization (≥ 1 night in the past 6 months)¥, n (%)					NA
Yes	–	–	–	320/4414 (7.0%)	

For peer review only

For peer review only - http://bmjopen.bmj.com/site/about/guidelines.xhtml

Emergency Department visit (in the past 6 months)[¥], n (%)<br>Yes	–	–	–	324/4414 (4.8%)	NA

Abbreviations: SD=Standard deviation, BMI=Body mass index, NA=Not applicable
Numbers may not add up to totals due to missing data.
[†]Self-reported diagnosis or medication use.
[¥]Only participants aged 60 years and above.
Statistical significance testing by chi-square (χ^2) test and one-way ANOVA with post-hoc Bonferroni test.

Figure 2 displays the graphical trends of the number of chronic conditions and multimorbidity of the study population by age groups (18–24, 25–34, 35–44, 45–54, 55–64, ≥ 65 years). The number of chronic conditions and multimorbidity increased linearly with age (p<0.001). The prevalence of any chronic condition increased from 27.9% in the youngest age group (18–24 years) to as high as 55.7% in the group of ≥ 65 years (Figure 2). Furthermore, multimorbidity and number of chronic conditions were observed to be more prevalent for individuals of female sex (p<0.001), except for participants with ≥ 6 chronic conditions, as demonstrated in Table 1 and Figure 3.

The results of multivariable logistic regression analysis assessing the association between socio-demographics, mental health and multimorbidity are shown in Table 3. Older age was associated with a higher likelihood of having multimorbidity (adjusted OR=1.05, 95% CI=1.05–1.05).

Table 3. Multivariable logistic regression modelling results of factors associated with multimorbidity (yes/no)

Parameter	Crude OR	Adjusted -OR	95% Confidence interval (CI)		*p*-value for adjusted-OR
			Lower bound	Upper bound	
**Age**	1.05***	1.05***	1.05	1.05	**<0.001**
**Sex**					**<0.001**
Male (Ref)		1			

For peer review only - http://bmjopen.bmj.com/site/about/guidelines.xhtml

Parameter	Crude OR	Adjusted -OR	95% Confidence interval (CI)		*p*-value for adjusted-OR
			Lower bound	Upper bound	
Female	1.33***	1.28***	1.17	1.40	
**Ethnicity**					0.356
Malay (Ref)		1			
Non-Malay	1.06	1.04	0.96	1.12	
**Education level**					**<0.001**
No formal education (Ref)		1			
Primary	0.63***	0.63***	0.53	0.74	**<0.001**
Secondary	0.66***	0.60***	0.51	0.70	**<0.001**
Tertiary	0.72***	0.65***	0.54	0.80	**<0.001**
**Employment status**					**<0.001**
Working/ Self-employed	0.30***	0.70***	0.60	0.82	**<0.001**
Student/Homemaker/ Unemployed	0.60***	0.87	0.74	1.02	0.077
Retiree (Ref)		1			
**Mental health**					0.816
No depressive symptoms (Ref)		1			
Depressive symptoms	0.96	1.01	0.91	1.13	

Abbreviation: Ref=Reference group, OR=Odds Ratio.
Diagnostic tests for complete case models: Nagelkerke R^2= 0.159.
* $p<0.05$, ** $p<0.01$, *** $p<0.001$.

In addition to older age, results from the multivariable logistic regression show that multimorbidity was associated with other socio-demographic factors, such as female sex, education level, and employment status (Table 3). Specifically, women are 1.28 times (95%

For peer review only - http://bmjopen.bmj.com/site/about/guidelines.xhtml

CI=1.17–1.40) more likely to experience multimorbidity compared to men. Similarly, having no formal education increased the odds of developing multimorbidity (primary education: adjusted-OR=0.63, 95% CI=0.53–0.74, secondary education: adjusted-OR=0.60, 95% CI=0.51–0.70; tertiary education: adjusted-OR=0.65, 95% CI=0.54–0.80). Working group was less likely to develop multimorbidity than retirees (adjusted-OR=0.70, 95% CI=0.60–0.82).

DISCUSSION

This population-based study provided evidence on the magnitude of multimorbidity and individual chronic conditions in a HDSS-scale community-dwelling general adult population. In addition, we identified various socio-demographic factors that could play a significant role in contributing to multimorbidity.

Prevalence of multimorbidity and individual chronic conditions

Using simple disease counts of 11 selected chronic conditions, we estimated the prevalence of multimorbidity at 28.5% in the overall study population. Musculoskeletal disorder, obesity, hypertension, and depressive symptoms are the most prominent physical and mental health conditions in this population. As expected, multimorbidity is more prevalent in older adults, affecting nearly half of the individuals aged 65 years and older (46.8%). However, it is worth noting that multimorbidity is affecting almost 10% of the young adults (18–34 years) and approximately one-third of the middle-aged adults (35–59 years), implying that multimorbidity is not just an issue of the elderly.

Extensive global and regional research has been conducted, showing substantial variability in the prevalence of multimorbidity. A systematic review and meta-analysis of 68 community-based studies worldwide by Nguyen and colleagues (2019) reported a prevalence

For peer review only

For peer review only - http://bmjopen.bmj.com/site/about/guidelines.xhtml

range of multimorbidity of 3.5% to 70% in 37 HICs, and 1% to 90% in 31 LMICs (8). However, the number of diseases used as cut-off points for disease counts, disease combination, age ranges, and definitions of multimorbidity differ widely across the studies. These inevitable heterogeneities, subsequently, generated inconsistent measures and prevalence of multimorbidity that hindered the comparability of findings between studies. Acceptance of a standard methodology will provide more clarity on the epidemiology of multimorbidity. The prevalence of multimorbidity in other South East Asian countries is very limited, and varies ethnically and geographically. An Indonesian community-based survey conducted among 9,438 adults aged 40 years and above found a higher prevalence of multimorbidity (35.7%) than that of our findings. Nevertheless, the prevalence of multimorbidity in our studies is similar but higher than in Singapore (26.2%) (24) as well as the few local studies in Malaysia (12, 13, 15-17), including the only three studies conducted by in-country researchers (15-17). The measures of these studies were constrained by the number of considered chronic conditions (i.e., < 10 diseases), relatively old data (i.e., 20 years ago), or only involved older cohorts (aged ≥ 56 years). Therefore, the present study has addressed recent calls for investigations in general and multi-ethnic population samples, assessing a broader range of chronic conditions characterised by real-world community-based large data comprising medical diagnoses, anthropometric measurements, psychological, and environmental risk factors.

Factors associated with multimorbidity

Our findings are in agreement with the known paradox and other studies that multimorbidity increases with age, as long-term diseases accumulate with age (10, 25). It is interesting to note that in our study population, older age only explained part of the augmented risk of

For peer review only - http://bmjopen.bmj.com/site/about/guidelines.xhtml

multimorbidity. We identified that other factors (sex, education level and employment status) are responsible for a substantial part of the association with multimorbidity. The inconsistency in sex differences and multimorbidity is not new, although most studies reported a higher prevalence of multimorbidity in women (26, 27). In this study, women were also more likely to be affected by multimorbidity. The findings are congruent with the literature in LMICs (15, 27, 28), but not with the findings of the Collaborative Research on Ageing in Europe (COURAGE) study conducted in Finland, Poland and Spain (26), the HICs. This discrepancy could be attributed to shared effects of biological, social, psychological, and environmental factors that generated inequalities in health status (29, 30). Education level appears to be associated with a higher risk of multimorbidity in our study. We found that individuals in general with no education had a higher likelihood of having multimorbidity than their counterparts. This is broadly in line with numerous other studies (25, 28, 31, 32). The impact of education on multimorbidity might be due to higher socio-economic status and healthier behaviours and knowledge of individuals with higher education than those with less-educated backgrounds (32). Employment status was associated with multimorbidity in the study population. Compared to retirees, working adults had a lower likelihood of developing multimorbidity. Age factor may potentially be underlying this relationship; this is not impossible when retirees are generally older than working individuals. Although highly prevalent among the participants, depressive symptoms did not appear to be a risk factor for multimorbidity in the study cohort. Contrarily, a systematic review suggested that the risk for depression was twice as great for people with multimorbidity compared to those without multimorbidity (33). The cross-sectional nature of our data prevents us from establishing the causal relationship between mental health and multimorbidity. This warrants further exploration in prospective cohort studies.

For peer review only - http://bmjopen.bmj.com/site/about/guidelines.xhtml

For peer review only

Strengths and limitations

The strengths of the present study include the use of a population-based dataset with high participation and completion rate (71.1%) that includes individuals of a broad range of ages, including those where chronic disease load begins to manifest. We have also captured a more comprehensive range of current and chronic conditions of ageing that have not been included in previous studies of multimorbidity. To the best of our knowledge, this is the first study investigating multimorbidity in a HDSS-scale real-world community-based general cohort in Malaysia. The regular surveillance of the large geographically defined population allowed for unique and detailed insight into epidemiological and health services changes. This includes the effects on individuals, households, and entire communities. The breadth of sophisticated measures available for use as covariates, spanning socio-economic and demographic domains, is a further strength of the current study, as it allowed a more thorough investigation of factors associated with multimorbidity than is usually attainable with medical records.

Nonetheless, the current research is not without limitations. First, causality cannot be assessed based on the nature of chronic condition data and hence cross-sectional study design. Future longitudinal investigation would allow an opportunity for causal inference. Second, the clinical diagnoses of medical conditions were self-reported by the participants, thus, may be susceptible to bias and inaccuracies that deviate from medical records. Nevertheless, self-report of diagnosis of chronic conditions has been widely used in epidemiological studies and proven valid (34). Third, the reports of chronic conditions were restricted to the list included in the interview. As such, the prevalence of multimorbidity reported may be underestimated. However, we have selected the survey rounds with the longest lists of chronic conditions possible in our longitudinal databases and have included all

For peer review only - http://bmjopen.bmj.com/site/about/guidelines.xhtml

relevant diseases for the assessment. Thus, we provided a detailed insight into multimorbidity in the study population. Fourth, given dementia is prevalent among older adults and people in this age group accounted for approximately 25% of the study sample, engaging in data collection of formal diagnoses of dementia is important and shall be considered in the definition of multimorbidity in the future. Lastly, it should be acknowledged that the surveys were conducted on participants who resided in semi-urban and rural areas. In this regard, certain groups of the population may be under-represented, such as those with higher socio-economic status, and patients who utilised private healthcare facilities centred around urban areas with more optimal disease control.

Implications for policy, practice, and future research

The knowledge and evidence deficiencies of the epidemiology of multimorbidity in Malaysia are now addressed. They are vital steps towards future research and sensible governance and policy development to prevent developing multimorbidity and tackle the growing problems multimorbidity brings. Our findings facilitate the identification of high-risk populations and suggest a scope for a prevention strategy focusing on reducing the development of multimorbidity. Given the high prevalence of multimorbidity and the expected increase in the future, the current single-disease-specific design of the health system should also be accompanied by strategies to address complexities associated with multimorbidity. Our findings suggest that the key to managing multimorbidity may be to strengthen a realistic and multidisciplinary approach that simultaneously targets health outcomes and socio-economic and demographics. The findings of this study could also be applicable for improving health outcomes and policy-making in other countries with social and economic developments comparable to that in Malaysia. We propose further research on the efficacy and

For peer review only - http://bmjopen.bmj.com/site/about/guidelines.xhtml

effectiveness of trials is critical to be tested and established in better-equipped scientific settings with trained research staff. Therefore, funding and initiatives are important to translate the research findings into real-world settings, while exploring cost-effectiveness as a dual strategy in a resource-limited setting. Identifying specific clusters of chronic diseases that could cause the most significant problems is beyond the scope of this study, albeit its importance. Further research on multimorbidity clusters alongside their risk factors is needed to clarify relevant mechanisms to facilitate the development of targeted interventions for patients.

CONCLUSION

The study shows that multimorbidity is common among adults of any age. The prevalence and risk of multimorbidity increased with age. Multimorbidity is also associated with sex, education level, and employment status, indicating that multimorbidity is not just an issue of the elderly. Besides contributing to the developing literature for multimorbidity in LMICs, this study highlights the importance of prioritising health policies, integrated care interventions, and clinical practice. All these should be oriented to implementing and delivering changes that include evidence-based components for effective preventative intervention and management of multimorbidity.

Contributors

Conceptualization: MMCT, MP, GMT, DM, TTS. Data Curation: MMCT, MRI. Formal Analysis: MMCT. Funding Acquisition: MP, GMT, DM, TTS. Investigation: MMCT, MP, GMT, PA, DR, TTS. Methodology: MMCT, MP, GMT, DM, PA, DR, TTS. Project Administration: MMCT. Resources: MMCT, MP, GMT, ZK, PA, DR, TTS. Software: MMCT, MP. Supervision: MMCT, MP, GMT, TTS. Visualization: MMCT. Writing – Original Draft Preparation: MMCT. Writing – Review & Editing: MMCT, MP, GMT, DM, MRI, EA, AÁMK, PA, DR, TTS.

Ethics approval

For peer review only - http://bmjopen.bmj.com/site/about/guidelines.xhtml

Ethics approvals were obtained from the Monash University Human Research Ethics Committee (MUHREC CF11/3663 – 2011001930 and 2013-3837-3646). All participants had provided written consent prior to data collection.

Funding

This work and publication of this article were supported by the Medical Research Council (UK Research and Innovation) grant (MUTUAL grant, King's College London, Grant number MR/T037423/1 awarded to MP and team). Funding for SEACO HDSS was provided by the research offices of Monash University in Australia and Malaysia (Faculty of Medicine, Nursing and Health Sciences; Jeffrey Cheah School of Medicine and Health Sciences; and Faculty of Arts).

Disclaimer

The funders had no role in study design and the execution of the study.

Data availability statement

Data is available on request from the corresponding authors, or by application to the SEACO, Jeffrey Cheah School of Medicine and Health Sciences, Monash University Malaysia.

Competing interests

The authors have declared that no competing interests exist.

Open Access

This is an Open Access article distributed in accordance with the Creative Commons Attribution (CC BY 4.0) license, which permits others to copy, redistribute, remix, transform and build upon this work for any purpose, provided the original work is properly cited, a link to the licence is given, and indication of whether changes were made. See: https://creativecommons.org/licenses/by/4.0/.

REFERENCES

1. World Health Organization (WHO). Multimorbidity: Technical series on safer primary care. Geneva: World Health Organization; 2016.
2. The Academy of Medical Sciences UK. Multimorbidity: A priority for global health research. Academy of Medical Sciences, London; 2018 April 2018.
3. Fortin M, Lapointe L, Hudon C, Vanasse A, Ntetu AL, Maltais D. Multimorbidity and quality of life in primary care: A systematic review. Health and Quality of life Outcomes. 2004;2(1):1-12.
4. Ryan A, Wallace E, O'Hara P, Smith SM. Multimorbidity and functional decline in community-dwelling adults: A systematic review. Health and Quality of Life Outcomes. 2015;13(1):1-13.
5. Gallo JJ, Hwang S, Joo JH, Bogner HR, Morales KH, Bruce ML, et al. Multimorbidity, depression, and mortality in primary care: randomized clinical trial of an evidence-based depression care management program on mortality risk. Journal of General Internal Medicine. 2016;31(4):380-6.

For peer review only - http://bmjopen.bmj.com/site/about/guidelines.xhtml

6. Lehnert T, Heider D, Leicht H, Heinrich S, Corrieri S, Luppa M, et al. Health care utilization and costs of elderly persons with multiple chronic conditions. Medical Care Research and Review. 2011;68(4):387-420.
7. Stirland LE, González-Saavedra L, Mullin DS, Ritchie CW, Muniz-Terrera G, Russ TC. Measuring multimorbidity beyond counting diseases: systematic review of community and population studies and guide to index choice. BMJ. 2020;368.
8. Nguyen H, Manolova G, Daskalopoulou C, Vitoratou S, Prince M, Prina AM. Prevalence of multimorbidity in community settings: A systematic review and meta-analysis of observational studies. Journal of Comorbidity. 2019;9:2235042X19870934.
9. van Oostrom SH, Gijsen R, Stirbu I, Korevaar JC, Schellevis FG, Picavet HSJ, et al. Time trends in prevalence of chronic diseases and multimorbidity not only due to aging: Data from general practices and health surveys. PlOS One. 2016;11(8):e0160264.
10. Jürisson M, Pisarev H, Uusküla A, Lang K, Oona M, Kalda R. Prevalence of chronic conditions and multimorbidity in Estonia: A population-based cross-sectional study. BMJ Open. 2021;11(10):e049045.
11. The World Bank IBRD IDA. World Bank Open Data 2021 [Available from: http://data.worldbank.org/.
12. Tey NP, Siraj SB, Kamaruzzaman SBB, Chin AV, Tan MP, Sinnappan GS, et al. Aging in multi-ethnic Malaysia. The Gerontologist. 2016;56(4):603-9.
13. Stubbs B, Vancampfort D, Veronese N, Kahl K, Mitchell A, Lin P-Y, et al. Depression and physical health multimorbidity: primary data and country-wide meta-analysis of population data from 190 593 people across 43 low-and middle-income countries. Psychological Medicine. 2017;47(12):2107-17.
14. Afshar S, Roderick PJ, Kowal P, Dimitrov BD, Hill AG. Global patterns of multimorbidity: a comparison of 28 countries using the world health surveys. Applied Demography and Public Health in the 21st Century: Springer; 2017. p. 381-402.
15. Hussin NM, Shahar S, Din NC, Singh DKA, Chin A-V, Razali R, et al. Incidence and predictors of multimorbidity among a multiethnic population in Malaysia: a community-based longitudinal study. Aging Clinical & Experimental Research. 2019;31(2):215-24.
16. Yap KH, Warren N, Allotey P, Reidpath DD. Chronic disease profiles of subjective memory complaints: a latent class analysis of older people in a rural Malaysian community. Aging & Mental Health. 2020;24(5):709-16.
17. Ghazali SS, Seman Z, Zainuddin NH, Omar MA, Sooryanarayana R, Ariaratnam S, et al. Prevalence and factors associated with multimorbidity among older adults in Malaysia: a population-based cross-sectional study. BMJ Open. 2021;11(10):e052126.
18. Partap U, Young EH, Allotey P, Soyiri IN, Jahan N, Komahan K, et al. HDSS profile: the South East Asia community observatory health and demographic surveillance system (SEACO HDSS). International Journal of Epidemiology. 2017;46(5):1370-1g.
19. World Health Organization (WHO). Body mass index - BMI Geneva: World Health Organization (WHO); 2019 [Available from: http://www.euro.who.int/en/health-topics/disease-prevention/nutrition/a-healthy-lifestyle/body-mass-index-bmi.
20. World Health Organization (WHO). Global Health Estimates 2016: Deaths by cause, age, sex, by country and by region, 2000-2016. Geneva, Switzerland: World Health Organization (WHO); 2018.
21. Henry JD, Crawford JR. The short-form version of the Depression Anxiety Stress Scales (DASS-21): Construct validity and normative data in a large non-clinical sample. British Journal of Clinical Psychology. 2005;44(2):227-39.

For peer review only - http://bmjopen.bmj.com/site/about/guidelines.xhtml

22. Lovibond PF, Lovibond SH. The structure of negative emotional states: Comparison of the Depression Anxiety Stress Scales (DASS) with the Beck Depression and Anxiety Inventories. Behaviour Research and Therapy. 1995;33(3):335-43.
23. Musa R, Fadzil MA, Zain Z. Translation, validation and psychometric properties of Bahasa Malaysia version of the Depression Anxiety and Stress Scales (DASS). ASEAN Journal of Psychiatry. 2007;8(2):82-9.
24. Low LL, Kwan YH, Ko MSM, Yeam CT, Lee VSY, Tan WB, et al. Epidemiologic characteristics of multimorbidity and sociodemographic factors associated with multimorbidity in a rapidly aging Asian country. JAMA Network Open. 2019;2(11):e1915245-e.
25. Blümel JE, Carrillo-Larco RM, Vallejo MS, Chedraui P. Multimorbidity in a cohort of middle-aged women: Risk factors and disease clustering. Maturitas. 2020;137:45-9.
26. Garin N, Koyanagi A, Chatterji S, Tyrovolas S, Olaya B, Leonardi M, et al. Global multimorbidity patterns: a cross-sectional, population-based, multi-country study. Journals of Gerontology Series A: Biomedical Sciences and Medical Sciences. 2016;71(2):205-14.
27. Hussain MA, Huxley RR, Al Mamun A. Multimorbidity prevalence and pattern in Indonesian adults: An exploratory study using national survey data. BMJ Open. 2015;5(12):e009810.
28. Jankovic J, Mirkovic M, Jovic-Vranes A, Santric-Milicevic M, Terzic-Supic Z. Association between non-communicable disease multimorbidity and health care utilization in a middle-income country: Population-based study. Public Health. 2018;155:35-42.
29. Goldstein JM, Langer A, Lesser JA. Sex differences in disorders of the brain and heart—A global crisis of multimorbidity and novel opportunity. JAMA Psychiatry. 2021;78(1):7-8.
30. Sharma P, Maurya P. Gender differences in the prevalence and pattern of disease combination of chronic multimorbidity among Indian elderly. Ageing International. 2022;47(2):265-83.
31. Johnson-Lawrence V, Zajacova A, Sneed R. Education, race/ethnicity, and multimorbidity among adults aged 30–64 in the National Health Interview Survey. SSM-Population Health. 2017;3:366-72.
32. Demirchyan A, Khachadourian V, Armenian HK, Petrosyan V. Short and long term determinants of incident multimorbidity in a cohort of 1988 earthquake survivors in Armenia. International Journal for Equity in Health. 2013;12(1):1-8.
33. Read JR, Sharpe L, Modini M, Dear BF. Multimorbidity and depression: A systematic review and meta-analysis. Journal of Affective Disorders. 2017;221:36-46.
34. Simpson CF, Boyd CM, Carlson MC, Griswold ME, Guralnik JM, Fried LP. Agreement between self-report of disease diagnoses and medical record validation in disabled older women: factors that modify agreement. Journal of the American Geriatrics Society. 2004;52(1):123-7.

Figures

Figure 1. Study sampling flowchart

Figure 2. Graphical representation of the prevalence of multimorbidity and number of chronic conditions of the study population by age groups (n=18,101)

For peer review only - http://bmjopen.bmj.com/site/about/guidelines.xhtml

599 **Figure 3. Graphical representation of the sex-disaggregated prevalence of multimorbidity**
600 **and number of chronic conditions of the study population**

For peer review only

For peer review only - http://bmjopen.bmj.com/site/about/guidelines.xhtml

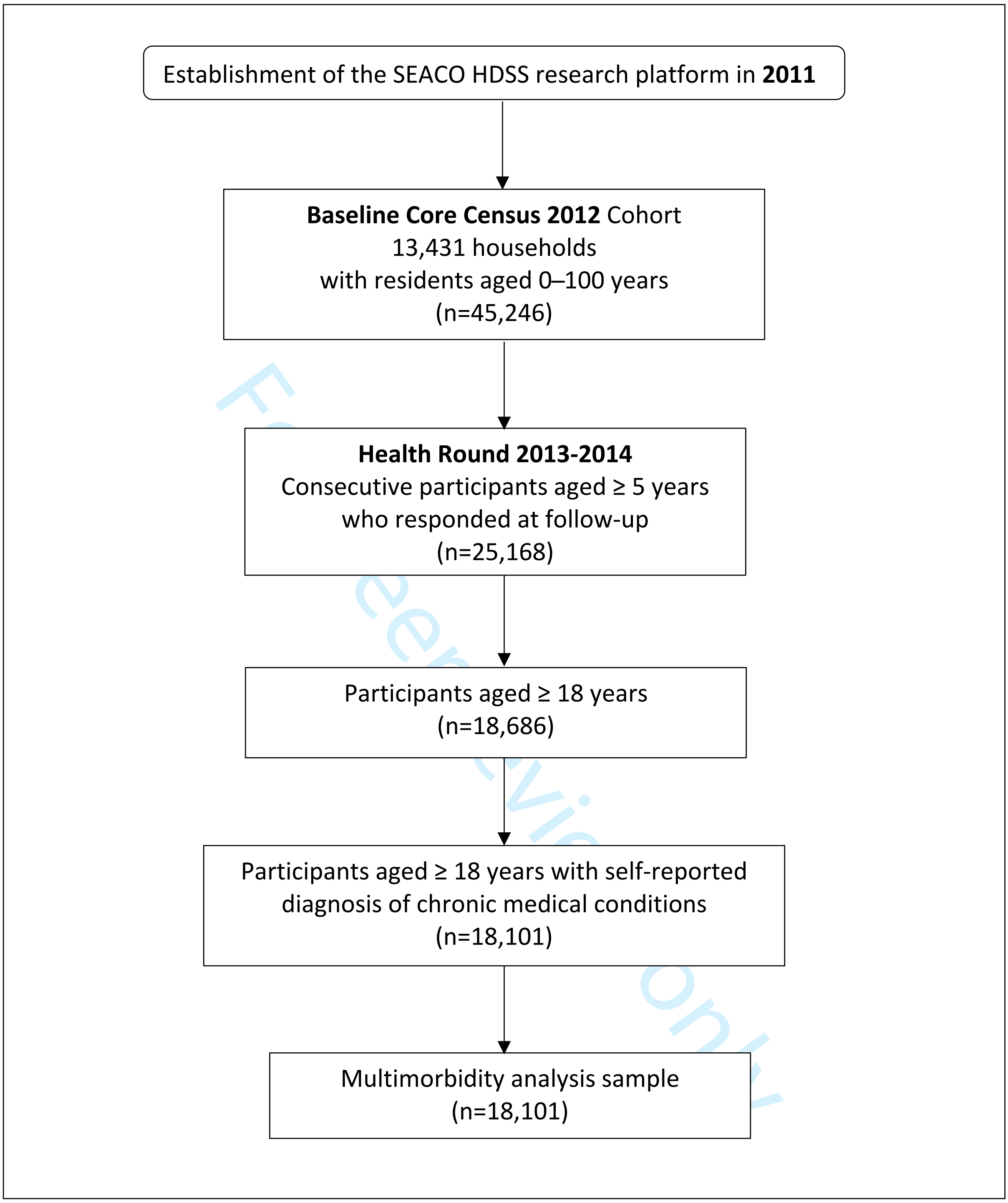

Figure 1 Study sampling flowchart

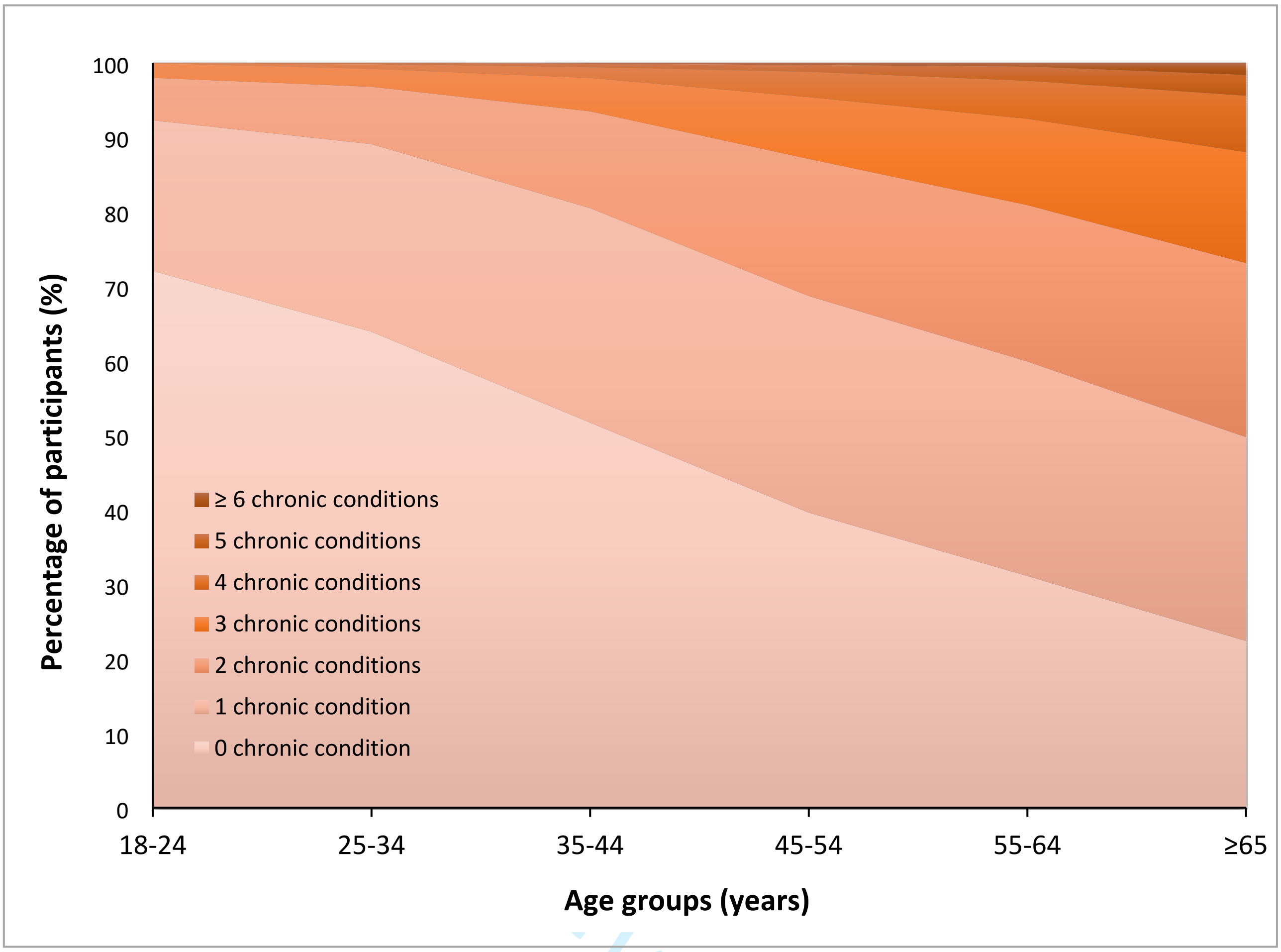

Figure 2 Graphical representation of the prevalence of multimorbidity and number of chronic conditions of the study population by age groups (n=18,101).

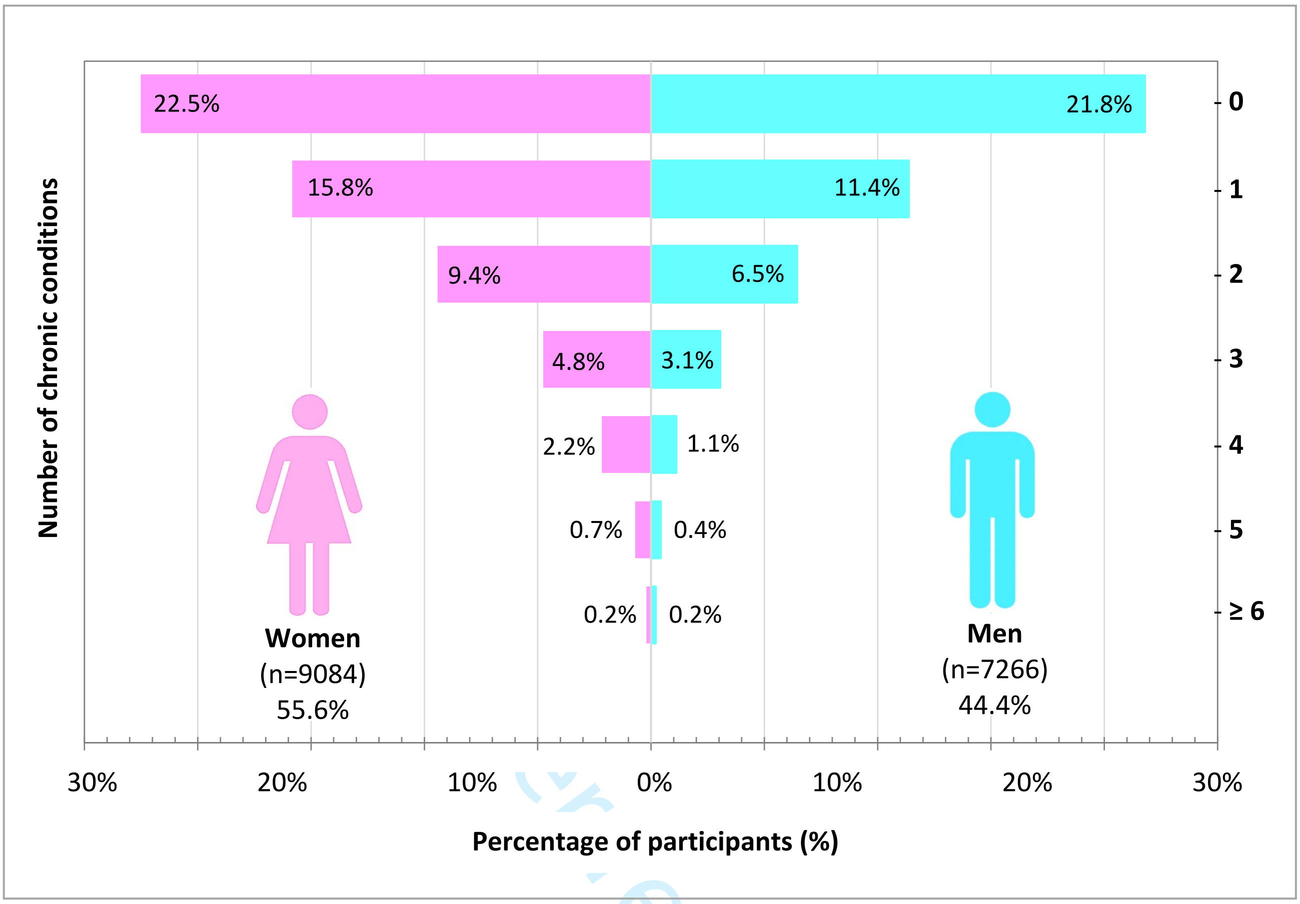

Figure 3 Graphical representation of the sex-disaggregated prevalence of multimorbidity and number of chronic conditions of the study population.

STROBE Statement—Checklist of items that should be included in reports of ***cross-sectional studies***

	Item No	Recommendation	Page No
**Title and abstract**	1	(*a*) Indicate the study's design with a commonly used term in the title or the abstract	1, 3
		(*b*) Provide in the abstract an informative and balanced summary of what was done and what was found	1, 3
**Introduction**			
Background/rationale	2	Explain the scientific background and rationale for the investigation being reported	3-5
Objectives	3	State specific objectives, including any prespecified hypotheses	5
**Methods**			
Study design	4	Present key elements of study design early in the paper	5
Setting	5	Describe the setting, locations, and relevant dates, including periods of recruitment, exposure, follow-up, and data collection	5, 6
Participants	6	(*a*) Give the eligibility criteria, and the sources and methods of selection of participants	6, 7
Variables	7	Clearly define all outcomes, exposures, predictors, potential confounders, and effect modifiers. Give diagnostic criteria, if applicable	8-10
Data sources/ measurement	8*	For each variable of interest, give sources of data and details of methods of assessment (measurement). Describe comparability of assessment methods if there is more than one group	8-10
Bias	9	Describe any efforts to address potential sources of bias	N/A
Study size	10	Explain how the study size was arrived at	7
Quantitative variables	11	Explain how quantitative variables were handled in the analyses. If applicable, describe which groupings were chosen and why	10
Statistical methods	12	(*a*) Describe all statistical methods, including those used to control for confounding	10
		(*b*) Describe any methods used to examine subgroups and interactions	10
		(*c*) Explain how missing data were addressed	N/A
		(*d*) If applicable, describe analytical methods taking account of sampling strategy	10
		(*e*) Describe any sensitivity analyses	N/A
**Results**			
Participants	13*	(a) Report numbers of individuals at each stage of study—eg numbers potentially eligible, examined for eligibility, confirmed eligible, included in the study, completing follow-up, and analysed	7, 10, 11
		(b) Give reasons for non-participation at each stage	7
		(c) Consider use of a flow diagram	7
Descriptive data	14*	(a) Give characteristics of study participants (eg demographic, clinical, social) and information on exposures and potential confounders	7-13
		(b) Indicate number of participants with missing data for each variable of interest	Footnotes of Tables 1 & 2

For peer review only - http://bmjopen.bmj.com/site/about/guidelines.xhtml

Outcome data	15*	Report numbers of outcome events or summary measures	14-16
Main results	16	(*a*) Give unadjusted estimates and, if applicable, confounder-adjusted estimates and their precision (eg, 95% confidence interval). Make clear which confounders were adjusted for and why they were included	15, 16
		(*b*) Report category boundaries when continuous variables were categorized	9, 10
		(*c*) If relevant, consider translating estimates of relative risk into absolute risk for a meaningful time period	N/A
Other analyses	17	Report other analyses done—eg analyses of subgroups and interactions, and sensitivity analyses	10
**Discussion**			
Key results	18	Summarise key results with reference to study objectives	16
Limitations	19	Discuss limitations of the study, taking into account sources of potential bias or imprecision. Discuss both direction and magnitude of any potential bias	18-20
Interpretation	20	Give a cautious overall interpretation of results considering objectives, limitations, multiplicity of analyses, results from similar studies, and other relevant evidence	16-20
Generalisability	21	Discuss the generalisability (external validity) of the study results	20-21
**Other information**			
Funding	22	Give the source of funding and the role of the funders for the present study and, if applicable, for the original study on which the present article is based	21

*Give information separately for exposed and unexposed groups.

Note: An Explanation and Elaboration article discusses each checklist item and gives methodological background and published examples of transparent reporting. The STROBE checklist is best used in conjunction with this article (freely available on the Web sites of PLoS Medicine at http://www.plosmedicine.org/, Annals of Internal Medicine at http://www.annals.org/, and Epidemiology at http://www.epidem.com/). Information on the STROBE Initiative is available at www.strobe-statement.org.

For peer review only
